# Supplementary material for: Visualizing the disordered nuclear transport machinery in situ
Source: Nature. 2023 Apr 26;617(7959):162–9. doi: 10.1038/s41586-023-05990-0 (PMC10156602; doi:10.1038/s41586-023-05990-0)
Supplement: Supplementary file 1 — This file contains Supplementary Text, Supplementary Figs. 1–21, Supplementary Tables 1–4 and Supplementary References. [file 41586_2023_5990_MOESM1_ESM.pdf]

nature portfolio

https://doi.org/10.1038/s41586-023-05990-0

Supplementary information

Visualizing the disordered nuclear transport machinery in situ

In the format provided by the authors and unedited

Visualising the disordered nuclear transport machinery *in situ*

M. Yu *et al.*

mailto: edlemke@uni-mainz.de; gerhard.hummer@biophys.mpg.de

This PDF file includes:

Supplementary Text
Supplementary Figs. 1 to 21
Supplementary Tables 1 to 4
Supplementary References (86-93)

Supplementary Text

Live-cell labelling and the pitfalls of in-cell FRET measurements when using highly overexpressed NUP98

We first co-transfected NUP98 plasmids with a single amber site ($NUP98^{221TAG}$) and OTO-GCE system, and performed live-cell labelling with various cell-permeable dyes (include Janelia Fluor 549-tetrazine (JF549-tz), Janelia Fluor 646-tetrazine (JF646-tz), silicon rhodamine-tetrazine (SiR-tz), and TAMRA-tetrazine (TAMRA-tz)) as described in the Methods (see the section about *cell culture, transfection, and labelling*). For cell-permeable dyes, we could hardly identify a clear nuclear envelope from the background signals (which can be observed in experiments where the ncAA is unreactive BOC and without any ncAA in Extended Data Fig. 2) unless we highly overexpressed NUP98.

We then expressed and labelled NUP98 constructs with double amber sites in live cells, using a mixture of JF549 and JF646 as the FRET dye pair at the molar ratio of 1:2. We measured the lifetime for a longer chain segment ($NUP98^{221TAG\text{-}482TAG}$, N_{res} = 261 aa) and a shorter chain segment ($NUP98^{221TAG\text{-}251TAG}$, N_{res} = 30 aa) in COS-7 cells. We observed a faster lifetime decay for the shorter chain segment, which indicates higher FRET (Extended Data Fig. 6a). The lifetime trend is in line with our results in permeabilized cells (see the comparison in Extended Data Fig. 6a and b). However, when we labelled the single-amber-mutant $NUP98^{221TAG}$ with the same FRET dye pair, we detected a lifetime change before and after acceptor photobleaching (Fig. 2c), which indicates intermolecular FRET (i.e., the FRET between different NUP98 molecules) under the elevated overexpression condition for live-cell labelling. This is detrimental to our quantitative analysis because we cannot extract an accurate root-mean-square inter-residue distance from a mixture of intramolecular and intermolecular FRET.

FLIM analysis methods

The fluorescence lifetime decays for the eighteen chain segments of NUP98 inside the NPC were extracted from the selected ROIs using the FLIM-FRET measurement pipeline described in the Methods (see the section about *FLIM-FRET for cell measurements*). The observed emission from the donor channels consists of three populations: donor-only population I_{Donly}, FRET population between donor and acceptor dyes I_{FRET}, and cellular background signal I_{bg}. The time-resolved fluorescence intensity in the donor channel can thus be described by

$$I(t) = I_{\mathrm{Donly}}(t) + I_{\mathrm{FRET}}(t) + I_{\mathrm{bg}}(t)$$

$$= \left\{ A_{\mathrm{D}} e^{-\frac{t}{\tau_D}} + A_{\mathrm{FRET}} \int_0^{\infty} \rho(r) e^{-\frac{t}{\tau_D} * [1 + \left(\frac{R_0}{r}\right)^6]} \mathrm{d}r + A_{\mathrm{bg}} \sum_{i=1}^{N} \alpha_i \, e^{-\frac{t}{\tau_{bg_i}}} \right\} \otimes IRF \qquad [15]$$

$$A_{\mathrm{D}} + A_{\mathrm{FRET}} + A_{\mathrm{bg}} = 1$$

where A_D, A_{FRET}, and A_{bg} are the species fraction, i.e., the initial intensities (at t = 0) for the three components before acceptor photobleaching, respectively, τ_D is the donor fluorescence lifetime in the absence of an energy transfer acceptor, $\rho(r)$ is the distribution of the inter-residue distance r that characterizes the conformational plasticity of the chain segment, R_0 is the Förster distance of

the dye pair, τ_{bg_i} is the lifetime characterizing the particular exponential components of the cellular background with the species fraction α_i, and IRF is the instrument response function. The ensemble lifetime of the cellular background was determined from approximately 150 cells expressing the amber-mutant-NUP98 in the presence of BOC, a nonreactive ncAA. The added-up lifetime decay was fitted with a three-exponential function with TauFit in PAM[68]. After acceptor photobleaching, the time-resolved fluorescence intensity in the donor channel can be described by

$$I'(t) = \left\{A_{\mathrm{D}} e^{-\frac{t}{\tau_D}} + A_{\mathrm{FRET}} e^{-\frac{t}{\tau_D}} + A_{\mathrm{bg}} \sum_{i=1}^{N} \alpha_i \, e^{-\frac{t}{\tau_{bg_i}}}\right\} \otimes IRF \qquad [16]$$

Assuming no photobleaching of the donor and background during the FLIM-FRET measurements before and after acceptor photobleaching, the difference in the intensity is calculated by subtracting Eq. 15 from Eq. 16,

$$I'(t) - I(t) = A_{\mathrm{FRET}} \left\{e^{-\frac{t}{\tau_D}} - \int_0^\infty \rho(r) e^{-\frac{t}{\tau_D} * [1+\left(\frac{R_0}{r}\right)^6]} \mathrm{d}r\right\} \otimes IRF \qquad [17]$$

where the signals from the donor-only population and the background were eliminated, and the difference originates only from the FRET population. Here the "FRET population" refers to the NUP98 chains specifically labelled with a FRET dye pair, i.e., a donor dye and an acceptor dye. After acceptor photobleaching, the corresponding FRET signal disappears. We distinguish 'the FRET population' from the donor-only population where the protein chain was specifically labelled with only donor dyes but no acceptor dyes.

The site-specific but random labelling method that we adopted here results in an inherent donor-only component (as it leads to donor-donor, donor-acceptor/acceptor-donor, and acceptor-acceptor populations), which decreases the contribution of the distinct decay kinetics of the FRET signal[43] (see below the section about *the effect of donor-only species on the fluorescence lifetime decays*). We note, that dual-site-specific labelling into the same protein would require two orthogonal synthetase pairs enabled by two orthogonal translating organelles and two orthogonal ultrafast inverse Diels-Alder click chemistries, which are not yet available. Furthermore, even when a dual-site-specific labelling method exists, one would also need to ensure that the two labelled sites are both always photophysically active. Thus, using a FLIM-based method that is robust to such effects facilitated our choice of methods.

To reduce the donor-only component, we optimized the stoichiometric ratio between donor and acceptor dye in the mixture. We found that donor : acceptor = 1:2 offered us a relatively large FRET population to increase our ability to extract the lifetime from FRET. It has been shown[86] that it is practical to recover correct values of distance distribution parameters if the FRET labelling ratio $L = A_{\mathrm{FRET}}/(A_{\mathrm{FRET}}+A_{\mathrm{D}}) \geq 0.5$, which was satisfied with our optimized stoichiometric labelling ratio. The fitting accuracy is further improved if the background (A_{bg}) is known, which we determined by performing acceptor photobleaching. In the following section on t*he effect of donor-only species on the fluorescence lifetime decays*, we further used a simple toy to show how the combination of FLIM-FRET and acceptor photobleaching increases robustness of the analysis.

We noticed that the signals in the donor channel photobleached during the FLIM-FRET measurements. To minimize such effects, we limited the measurement time per cell to 5 min.

Furthermore, we corrected the intensity difference described in Eq. 17 as $I'(t)$-$\sigma I(t)$, with the correct factor σ determined experimentally to compensate for the photobleaching. Next, we calculated $I'(t)$-$\sigma I(t)$ for the measured 18 mutants. For each mutant, we normalized the intensity profile after the acceptor photobleaching, using the same normalization factor for the corresponding intensity profile before the acceptor photobleaching. As shown in Fig. 2e, we could qualitatively compare different mutants, where a bigger difference in the plot means a higher FRET efficiency and shorter R_E.

Note that the plots in main Fig. 2e report on the FRET population (i.e., the protein chain specifically labelled with a FRET dye pair as we defined above), but they are not the donor lifetime curves of the FRET signal $I_{FRET}(t)$. In order to extract the FRET signal from the ensemble donor lifetime curves in Extended Data Fig. 5a, we fit the lifetime curves with Eq. 15. In Extended Data Fig. 5b, we present the donor lifetime curves of the FRET signal $I_{FRET}(t)$ after subtracting the fitted donor-only $I_{Donly}(t)$ and fitted background signals $I_{bg}(t)$, where more pronounced differences between mutants can be seen.

The effect of donor-only species on the fluorescence lifetime decays

In our cellular system, we always have donor-only species caused by the site-specific but random labelling strategy and any non-specific sticking of dyes. The donor-only population will partially mask the FRET signals. That is why the difference in fluorescence lifetime for various FRET pairs in Extended data Fig.5a is small but clearly detectable if enough signal is accumulated over many cells.

To illustrate the effect of donor-only species on the measured fluorescence lifetime, we exemplarily detail here a simple toy model calculation with only two species, donor-only population, and FRET population. Let us assume a scenario in which the root-mean-square inter-residue distance R_E ranges from 40 Å to 160 Å, such that the corresponding FRET efficiency would change from ~0.95 to ~0.15 if we assume a Gaussian chain model. Now if we dope such a system with more and more donor-only population by increasing the donor-only fraction from 10% to 50% (i.e., the FRET population decreases from 90% to 50%), we can clearly see that the lifetime differences for various R_E become narrower (see Supplementary Fig. 1). The in-cell fluorescence signals can be further complicated by cellular autofluorescence and scattering. Though the existence of the donor-only population would 'hide' the FRET signal to some extent, we can still observe a clear trend among different mutants as shown in both time domain (Extended data Fig. 5a) and frequency domain (Fig. 2f), proving the robustness of our in-cell FLIM measurement pipeline.

By combining the FLIM measurements with the acceptor photobleaching method, we could unveil the signals of FRET population hidden by the donor-only population and show more pronounced differences among different mutants (Fig. 2e) by mathematically cancelling out signals from donor-only population and cellular background (see Eqs. 15-17 above in Supplementary Text). For example, in Supplementary Fig. 2 we present the result when performing the acceptor photobleaching procedure in the simple toy model system. As mentioned above, this turns Eq. 15 into Eq. 16 and enables us to calculate the time-resolved intensity

difference between after and before acceptor photobleaching, $I'(t)$-$I(t)$ using Eq. 17. As shown in Supplementary Fig. 2, we only see the difference from the FRET population, no matter how much donor-only population we initially have in the system before acceptor photobleaching. Thus, combining acceptor photobleaching with FLIM-FRET helps to increase the accuracy of our analysis.

In the future, it would be highly desired to develop an advanced GCE system where two types of noncanonical amino acids can be incorporated specifically at two sites in one protein which can be labelled ultrafast with donor and acceptor dyes with different chemical handles, respectively. In this way, we could avoid the donor-only population caused by site-specific but random labelling strategy. Furthermore, developing dyes with higher fluorogenecity would also help to reduce the donor-only population caused by non-specific sticking of dyes.

Comparison between a Gaussian chain model with a static model

The FRET efficiency E=1/(1+(r/R_0)^6) is commonly used to describe a static model (e.g., dsDNA) with a fixed inter-residue distance r. However, for an intrinsically disordered protein that populates an ensemble of rapidly interconverting conformations, we cannot use the above equation[45]. By combining Eqs. 12 and 13 in the Methods, we use the root-mean-square inter-residue distance, simply referred as R_E, derived from a Gaussian chain model to describe such a conformational ensemble. Note that the R_E derived from a Gaussian chain model cannot be directly compared to the R_E in a static model. For example, in Supplementary Fig. 5a, we plot E versus R_E for R_0 = 60 Å (the Förster distance for a commonly used FRET dye pair of Alexa 488 and Alexa 594), and R_0 = 77 Å (the FRET distance of the FRET dye pair of AZDye 594 and LD655 optimized for this work) in a static model (i.e., E=1/(1+(R_E/R_0)^6) or in a Gaussian chain model (Eqs. 12 and 13). For R_0 = 77 Å, the Gaussian chain model shows a broader distribution of R_E against E, compared to a static model. By plotting the 1st derivative of E versus R_E (Supplementary Fig. 5b), one can compare the sensitive range of R_E, where the Gaussian chain model tails towards longer distances compared to a static model.

Fitting the scaling law for NUP98 FG in the NPC

To extract the polymer scaling law for the eighteen chain segments of NUP98 inside the NPC, we used two fitting methods, including global fitting and mutant-by-mutant fitting.

We first performed global fitting on the ensemble lifetime decays. To reduce the number of parameters to fit, we adopted the Gaussian chain model (Eq. 13 of the main text), arguably the simplest and most cited model to describe the distribution of the inter-residue distance with least free fitting parameters, and embedded it into the self-written code adapted from the TauFit function of PAM[68]. We acknowledge that more complex and maybe even better model exist, but at the cost of introducing more variables that would reduce the robustness of the fit. By substituting the polymer scaling law, which is given by

$$R_E = \rho_0 N_{\text{res}}^{v} \qquad [18]$$

and Eq. 13 of the main text into Eq. 15, the lifetime decay of each mutant could be directly expressed with the apparent scaling exponent v, prefactor ρ_0, and the number of amino acid residues between the double-labelled sites N_{res}.

In the global fitting of the FLIM-FRET lifetime decays before acceptor photobleaching, v and ρ_0 were set as global parameters to be fitted. Förster distance R_0, the background lifetime $\sum_{i=1}^{N} \alpha_i \, e^{-\frac{t}{\tau_{bg_i}}}$, the fraction of donor-only A_{D} the donor lifetime τ_D and the fraction of background A_{bg} were experimentally determined independently and fixed as global parameters. Specifically, $\sum_{i=1}^{N} \alpha_i \, e^{-\frac{t}{\tau_{bg_i}}}$ was determined by fitting the background lifetime profiles in control measurements using the non-reactive ncAA Lys-BOC with multi-exponential decays. τ_D and A_{bg} were determined by fitting the lifetime decays after acceptor photobleaching with Eq. 16. A_{D} was determined by fitting the lifetime decays before acceptor photobleaching for the shortest mutants (i.e., $\mathrm{NUP98}^{221\mathrm{TAG}\text{-}251\mathrm{TAG}}$ and $\mathrm{NUP98}^{221\mathrm{TAG}\text{-}283\mathrm{TAG}}$) with Eq. 15, because their FRET signals are most distinguishable from the donor-only signals.

The error in even simpler FRET measurements has recently been detailed in community-wide studies[72]. In our work, biological heterogeneity due to e.g., cell-to-cell difference in signal-to-noise ratio is arguably the largest contributor to experimental uncertainty, which is best obtained by sampling large cell numbers.

To estimate the biological heterogeneity, we employed the bootstrap resampling method for each mutant and added up the lifetime decays of sampled cells (n = approximately 100 for each mutant). We then globally fitted the lifetime decays for all mutants (in total approximately 2000 cells) using the Maximum Likelihood Estimator (MLE) provided in the PAM package[68]. We repeated the resampling and global fitting procedure 50 times. The apparent scaling exponent was extracted as $v = 0.56 \pm 0.03$ Fig. 3). Here the error obtained from the bootstrap analysis mainly describes the error of the mean due to biological heterogeneity.

In the mutant-by-mutant fitting, we first extracted R_{E} of each mutant and then fitted R_{E} versus N_{res} to obtain the apparent scaling exponent for approximately 100 cells per mutant. Specifically, for each mutant, we first fitted the lifetime decay after acceptor photobleaching with Eq. 16 and extracted τ_D and A_{bg}. Then the obtained τ_D and A_{bg}, together with R_0, $\sum_{i=1}^{N} \alpha_i \, e^{-\frac{t}{\tau_{bg_i}}}$, A_{D}, were used to fit the lifetime decay before acceptor photobleaching with Eq. 15 using MLE to extract R_{E} for that mutant. We also estimated the error of each mutant with the bootstrap analysis. After determining the average R_{E} for all the mutants, we fitted R_{E} versus N_{res} with Eq. 18 and obtained the 95% confidence interval of the apparent scaling exponent as $v = 0.55 \pm 0.05$ (Supplementary Fig. 8). In contrast to the global fit, the mutant-by-mutant fit does not maximize the use of all available data at once, and thus we see this larger error as a much more conservative estimate. It is encouraging though, that the means of both methods are within 0.01, giving us confidence that v is clearly different from the collapsed state of purified NUP98 on a single-molecule level in solution and also above the theta-solvent point of $v = 0.5$, entering the good solvent regime ($v > 0.5$).

The self-avoiding walk (SAW) model has been proposed as a better descriptor for protein chains in good solvent. Encouraged by the fit with the Gaussian chain model, we performed a global fitting of our experimental data in the NPC also to the SAW-ν model by Zheng et al.[48]

$$\rho(r) = A\frac{4\pi}{\sqrt[2]{\langle r^2\rangle}}\left(\frac{r}{\sqrt[2]{\langle r^2\rangle}}\right)^{2+g} e^{-B\left(\frac{r}{\sqrt[2]{\langle r^2\rangle}}\right)^{\delta}} \qquad [19]$$

where $\sqrt[2]{\langle r^2\rangle} \equiv R_E$. A and B are constants determined by the normalization condition $\int_0^\infty \rho(r)dr = 1$ and $\int_0^\infty \rho(r)r^2dr = {R_E}^2$. The exponents in the SAW-ν model satisfy $\delta = 1/(1 - \nu)$, and $g = (\gamma - 1)/\nu$ with $\gamma \approx 1.1615$. As closure relation for proteins, the SAW-ν model of Zheng et al[48] uses Eq. 18 with ρ_0 = 0.55 nm (which is estimated for proteins in good solvent[48]). By performing the global fitting with Eqs. 18 and 19, we obtained the scaling exponent as $\nu = 0.56 \pm 0.001$. The perfect agreement with $\nu = 0.56 \pm 0.03$ obtained from our analysis using Gaussian chains shows the robustness of our scaling exponent for different polymer models and further strengthens our analysis.

<u>Fitting the scaling law of liquid-like NUP98 FG condensates formed in vitro from purified NUP98</u>.

To extract R_E of different mutants from the lifetime decays of the NUP98 FG condensates *in vitro*, a similar fitting pipeline as for in cell measurements was applied with some modifications. Since the phase-separated condensates demonstrated liquid-like behaviour at the early stage (as we measured for the first 5 min), where the fluorescence could recover after photobleaching, we could not perform the acceptor photobleaching to estimate the background signal ratio as we did for the cell measurements. Therefore, we first measured the lifetime profile for the condensates formed by the unlabelled NUP98 using the differential interference contrast (DIC) module to check the focusing plane. We then measured the lifetime decay for the unlabelled NUP98 mixed with the donor-only labelled sample with the same donor concentration as for the FRET samples and fitted the decay curve with Eq. 16 to determine τ_D and A_{bg}. After that, we performed the Gaussian chain model fitting of the lifetime curves for the FRET samples and extracted R_E for each mutant in the FG condensates. The FLIM-FRET measurements for the FG condensates were repeated 5 times for each mutant. We note, that in contrast to the in-situ measurements, we saw lifetime changes over time for the FG condensates in vitro, in line with fact that droplets molecularly age from a liquid-like to a hydrogel-like state, likely accompanied by molecular changes inside the droplets leading to heterogeneities[26]. By limiting ourself to the first 5 min after droplet formation, such effects could be reduced but likely not eliminated. Shorter measurement times were not eastly achievable due to the need to collect enough photons. Despite these complications, we were able to fit R_E versus N_{res} with Eq. 18 and extracted the 95% confidence interval of the apparent scaling exponent as $\nu = 0.56 \pm 0.04$ (main Fig. 3), in line with good solvent conditions. We also measured the scaling law for *in vitro* NUP98 FG droplets with the GLEBS domain (Extended Fig. 10d). While the prefactor might be more sensitive to heterogeneity in the gels, we also here recovered a scaling exponent in line with good solvent conditions of $\nu = 0.60 \pm 0.03$.

Comparison of intensity-based FRET versus lifetime-base FRET analysis at the single-molecule level.

While at the ensemble level, it is not possible to directly extract quantitative R_E values from measured FRET efficiencies, at the single-molecule level this is possible, because the donor-only fraction is directly detectable, which is widely established as a gold standard[72].

To verify our lifetime fitting algorithm, we extracted the fluorescence intensity profiles of the detected bursts from smFRET measurements dataset of the purified FRET labelled and highly diluted (pM concentrations) NUP98 in PBS *in vitro* (Extended data Fig. 8). We then performed the lifetime fitting for each mutant with Eqs. 13 and 15. The correlation of the R_E extracted from the lifetime fitting versus the R_E, obtained from the smFRET analysis described in the Methods (see section about *single-molecule FRET measurements*), is shown in Supplementary Fig. 4 with coefficient of determination R^2 = 0.96, validating our approach. By fitting R_E obtained from smFRET versus N_{res} with Eq. 18, we extracted the 95% confidence interval of the apparent scaling exponent as $\nu = 0.29 \pm 0.01$.

Flory-Huggins theory

We used the mean-field Flory-Huggins (FH) model[87–89] to estimate the phase-coexistence curve and phase diagram from condensate simulations. The free energy of mixing per monomer is given by

$$\frac{\Delta\bar{F}_{mix}}{k_B T} = \frac{\phi}{N}\ln\phi + (1-\phi)\ln(1-\phi) + \chi\phi(1-\phi) \qquad [20]$$

where ϕ is the polymer volume fraction, N = 499 is the number of monomers per chain, and χ is the FH parameter quantifying the interaction energy per monomer. The first two terms account for the entropy of mixing chains and solvent, respectively, and the third term accounts for the enthalpy of mixing. For the interaction strength larger than the critical interaction strength, $\chi > \chi_c = \frac{1}{2} + N^{-1/2}$, the system separates into two coexisting dilute and dense phases with volume fractions of $\Phi'_{\tilde{\epsilon}}$ and $\Phi''_{\tilde{\epsilon}}$, respectively. The chemical potential of the system is determined by

$$\frac{\mu}{k_B T} = \frac{1}{k_B T}\frac{\partial\Delta\bar{F}_{mix}}{\partial\phi} = \frac{1}{N}\ln\phi - \ln(1-\phi) + \frac{1}{N} - 1 + \chi(1-2\phi) \qquad [21]$$

By setting the chemical potentials of the dilute and dense phases equal, and solving for χ, one finds

$$\chi(\tilde{\epsilon}) = \frac{\frac{1}{\mathrm{N}}\log\left(\frac{\Phi''_{\tilde{\epsilon}}}{\Phi'_{\tilde{\epsilon}}}\right) + \log\left(\frac{1-\Phi'_{\tilde{\epsilon}}}{1-\Phi''_{\tilde{\epsilon}}}\right)}{2(\Phi'_{\tilde{\epsilon}} - \Phi''_{\tilde{\epsilon}})} \approx A\tilde{\epsilon} + B \qquad [22]$$

We find that for the volume fractions $\Phi'_{\tilde{\epsilon}}$ and $\Phi''_{\tilde{\epsilon}}$ observed in the simulations, $\tilde{\epsilon}$ and χ are linearly related to a good approximation (Supplementary Fig. 10). To interpret our simulation data in the FH model, we thus determined the volume fraction of each phase using $\Phi_{\tilde{\epsilon}} = c/\bar{\rho}$ where $\bar{\rho}$ =1263 mg/mL is the ratio of molar mass and molar volume of an isolated NUP98 FG chain (1-499) in aqueous solution obtained at α = 0.7 in the Martini model[79]. For each set of ($\tilde{\epsilon}$, $c^{\tilde{\epsilon}}_{\text{dilute}}$, $c^{\tilde{\epsilon}}_{\text{dense}}$) parameters, we used Eq. 22 to determine the corresponding value of χ. Supplementary Fig. 10

shows the linear relation between the $\tilde{\epsilon}$ and χ. According to the FH model for a chain of $N = 499$ residues, the critical cohesive interaction strength is then $\tilde{\epsilon}_c = 0.417$.

Molecular dynamics simulations of NUP98 condensates

With a coarse-grained bead-spring polymer model[53] parametrized to match the extension of single NUP98 FG chains(1-499) and the phase behaviour of NUP98 FG-chains, we could sample their large-scale motions and condensate formation. In simulations of 500 chains contained in an elongated box, we found that condensation occurs above a critical interaction strength of $\tilde{\epsilon_c} \approx 0.417$ (Extended data Fig. 10a and b). The phase coexistence line in the plane of concentration (c) and cohesive strength ($\tilde{\epsilon}$) is captured well by the Flory-Huggins (FH) mean-field model (Extended data Fig. 10c). For $\tilde{\epsilon} = 0.44$, the calculated concentrations of the dense and dilute phase (190.6 ± 8.7 mg/mL and 0.98 ± 0.80 mg/mL) closely match those reported for NUP98-FG and an engineered 12mer GLFG (0.3 - 0.5 mg/mL[27,54] and 175 mg/mL[54], respectively). Compared to single chains (Supplementary Fig. 9c), the chains in the condensate (Extended data Fig. 10d) are more extended with only a weak dependence of the extension on $\tilde{\epsilon}$. The interactions with other chains in effect mimic "good-solvent" conditions for the individual polymers, akin to the Flory hypothesis for polymer melts. Compared to the chains in the NPC (Fig. 4 and Supplementary Fig. 11), the chain distances are nearly insensitive to the interaction strength in the condensate, i.e., for $\tilde{\epsilon} > 0.42$ (Extended data Fig. 10d).

Coarse-grained model of NPC with explicit solvent

We investigated a possible effect of solvent on the configurations of the FG-NUPs by adding explicit solvent particles to the homopolymer model I (see Supplementary Table 2). We subcategorized protein residues (p) into scaffold residues (p_{sc}) and FG residues (p_{FG}), and we included in addition membrane particles (m) and solvent particles (s). The interaction between solvent particles and protein residues was modelled by the Weeks-Chandler-Andersen (WCA) repulsive potential[90]. The potential energy of the system was given by

$$U = U_{LJ} + U_{FENE} \tag{23}$$

$$= 4k_BT \sum_{i<j, r_{ij}<r_c^{\alpha}, i,j\in\{p,m,s\}} \tilde{\epsilon}_{ij} \left[\left(\frac{\sigma_{ij}}{r_{ij}}\right)^{12} - \left(\frac{\sigma_{ij}}{r_{ij}}\right)^{6} \right]$$

$$+ 4k_BT \sum_{\langle i,j=i+1\rangle, i,j\in p, r_{ij}<2^{\frac{1}{6}}\sigma} \left[\left(\frac{\sigma}{r_{ij}}\right)^{12} - \left(\frac{\sigma}{r_{ij}}\right)^{6} + \frac{1}{4} \right]$$

$$- \sum_{\langle i,j=i+1\rangle,\ i,j\in p} 0.5k_{FE}R_{FE}^2 \ln\left[1 - \left(\frac{r_{ij}}{R_{FE}}\right)^2\right]$$

The non-bonded interactions between particles i and j in the categories of FG-proteins, membrane, and solvent particles are modelled by LJ potentials, whose strength $\tilde{\epsilon}_{ij}$, length σ, and cut-off values r_c^{α} are listed in Supplementary Table 4. Bonds between neighbouring beads along the FG-proteins are described by the FENE potential with $k_{\mathrm{FE}} = 30k_{\mathrm{B}}T$ and $R_{\mathrm{FE}}=1.5\sigma$.

To model the membrane of the nuclear envelope, we first built a 100x100 nm^2 coarse-grained POPC lipid bilayer patch using python script insane.py[91,92]. We used a small area per lipid of 0.3 nm^2 to prevent solvent penetration into the membrane envelope (command: *insane.py -l POPC -x 100 -y 100 -z 100 -a 0.3 -o bilayer.gro*). Then, a half-toroidal membrane pore was generated with the BUMPy software[91] using the flat bilayer as membrane input (command: *bumpy.py -s double_bilayer_cylinder -f bilayer.gro -z 10 -g l_cylinder:10 r_cylinder:430 r_junction:120 l_flat:1920*). To minimize computational cost, we only kept the phosphate groups of the bilayer. Both the NPC scaffold residues and the membrane particles were fixed during the simulations, i.e., their equations of motion were not integrated. In order to avoid solvent and FG particles crossing the membrane, a LJ potential with large repulsive range was used (see Supplementary Table 4). We used a Langevin thermostat with damping coefficient 10τ to keep the temperature at $k_B T = 1$. The time step and averaging procedure were identical to the homopolymer system. The total simulation time for condensate and NPC simulations were 200,000 τ and 80,000 τ, respectively.

In the NPC model with explicit solvent, the extension of NUP98 follows the FRET measurements at an interaction strength of $\tilde{\epsilon} = 0.42$ again close to the critical value $\tilde{\epsilon}_c \approx 0.41$. The phase boundaries of implicit and explicit solvent models are compared in Supplementary Fig. 12. a slight shift in the critical value compared to implicit solvent, $\tilde{\epsilon}_c \approx 0.41$, is expected as solvent-monomer interactions enter the Flory parameter χ. We thus find near-critical conditions in the NPC both with implicit and explicit solvent (Fig. 4 and Extended Data Fig. 10).

Stickers-and-spacers-type models

Motivated by the stickers-and-spacers model of liquid-liquid phase separation[59], we accounted for possible effects of sequence heterogeneity in the FG-NUPs by distinguishing between “sticker” beads with strong mutual interactions and spacer beads with weak interactions (Supplementary Figs. 14-17). In the FYW-stickers model, all aromatic residues (Phe, Tyr, Trp) were defined as stickers. The simulation setup and parameters were otherwise identical to the those of the homopolymer model. With weak spacer-spacer and sticker-spacer interactions fixed at $\tilde{\epsilon}_{ij}$, = 0.3 ($i \in \{\text{spacers}\}, j \in \{\text{spacers, stickers}\}$), we varied the sticker-sticker interaction strength and matched the resulting NUP98 distances to the fluorescence measurements in the NPC (Supplementary Fig. 17). The resulting effective sticker-sticker interaction strength $\tilde{\epsilon}_{FYW} = 3.5$ is again close to the critical value in the phase diagram of FG-NUP98 condensate formation (Supplementary Fig. 16). Similarly, for isolated NUP98 chains we obtained the best match to the experiments in the collapsed regime (Supplementary Fig. 15). The main conclusions are thus unaltered when going from a homopolymer model to the FYW-stickers heteropolymer model.

Distribution of inter-residue distances

We also examined the distributions of the inter-residue distances between the NUP98 N-terminus and the anchoring point on the NPC scaffold, which follow theoretical predictions[93] for isolated chains, with a slight tendency to somewhat more extended states in the NPC (Supplementary Fig. 21).

Supplementary Figures

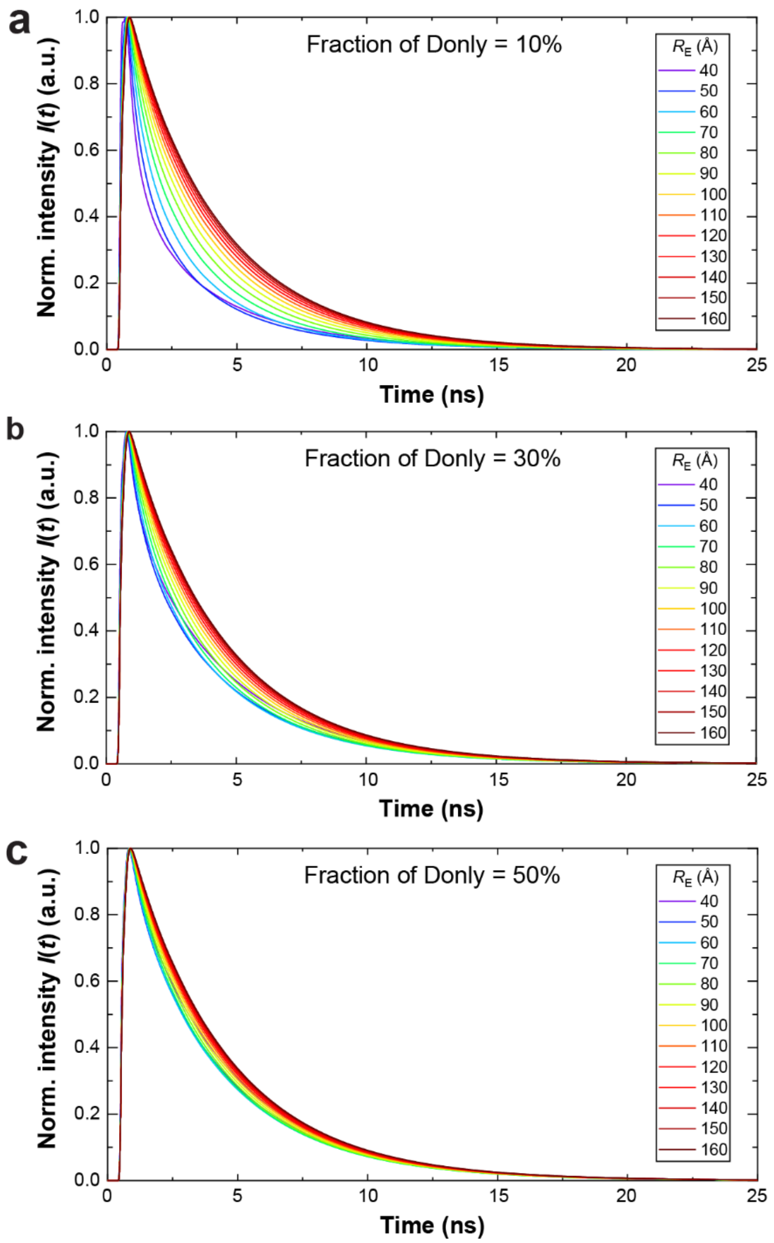

Supplementary Fig. 1 A simple toy model calculation to illustrate the effect of donor-only populations. In a simple model system with mixed donor-only population and FRET population, calculated fluorescence lifetime decays using Eq. 15 with root-mean-square inter-residue distance R_E ranging from 40 Å to 160 Å with **(a)** 10%, **(b)** 30%, and **(c)** 50% fractional population of donor-only (Donly). With increasing donor-only fraction, the different distances are harder to resolve.

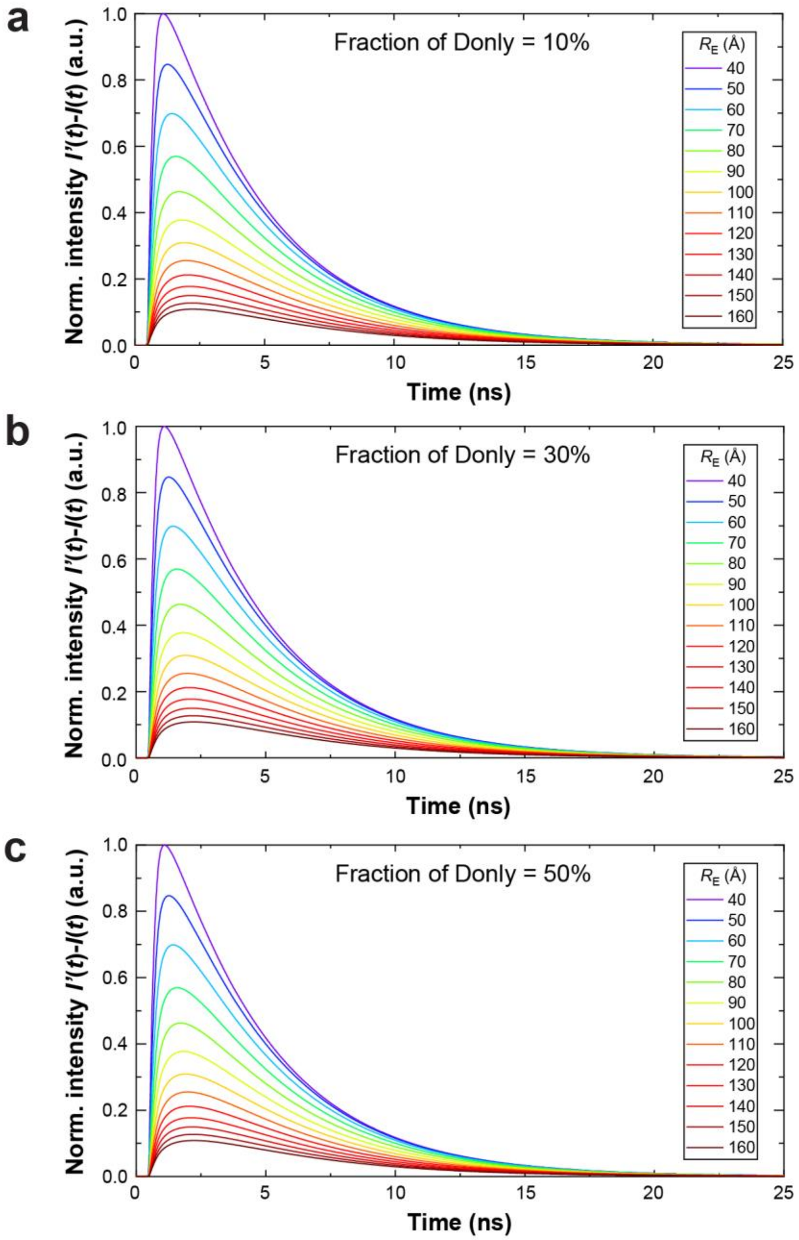

Supplementary Fig. 2 A simple toy model calculation to illustrate the effect of donor-only population when using FLIM-FRET combined with acceptor photobleaching method. Calculated differences using Eq. 17 in donor fluorescence intensity after and before acceptor photobleaching, $I'(t)$-$I(t)$, with root-mean-square inter-residue distance R_E ranging from 40 Å to 160 Å in the same model system as in Supplementary Fig. 1 with **(a)** 10%, **(b)** 30%, and **(c)** 50% fractional population of donor-only (Donly) before the acceptor photobleaching. Compared to the curves shown in Supplementary Fig. 1, here the curves for the different distances are better resolved from each other.

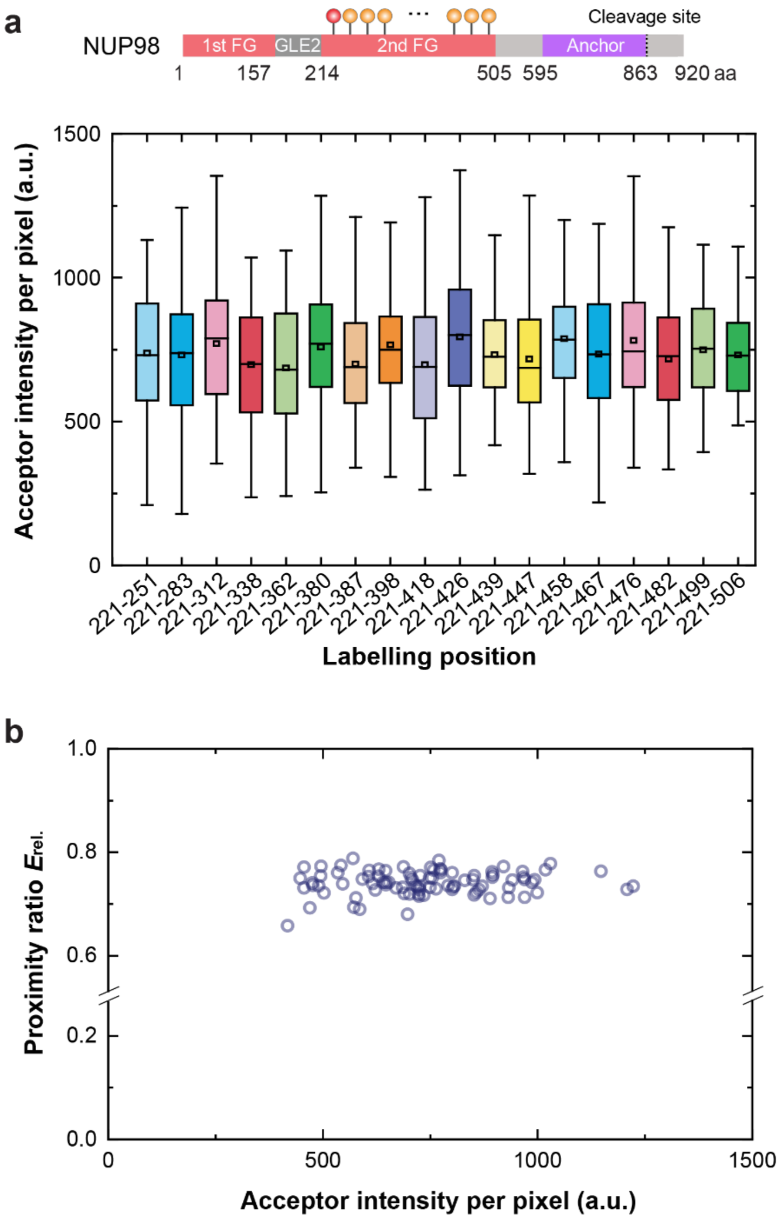

Supplementary Fig. 3 Selecting cells with similar expression levels by checking the acceptor intensity per pixel (excited by 660 nm laser). To ensure that cells with similar expression levels and not highly overexpressed mutant NUP98 were chosen (which typically leads to large visible aggregates), we always checked the acceptor intensity per pixel (excited by 660 nm laser) to estimate the expression level of the mutant NUP98 before we started the FRET measurements. **(a)** For all 18 double-labelled mutants, the distributions of the acceptor intensity per pixel for the selected cells are on a similar level (n = approximately 100 cells for each mutant). The box limits represent the range between the first and third quartiles for each mutant, the centre lines show the median, the central square shows the mean, and the ends of the whiskers extend to 1.5× the interquartile range. **(b)** All selected cells/mutants were checked for the presence of intermolecular FRET. Here shown is representatively a plot of relative FRET efficiency ($E_{rel.}=I_A/I_A+I_D$) versus acceptor intensity per pixel excited by 660 nm laser for NUP98$^{221TAG\text{-}439TAG}$. No correlation between $E_{rel.}$ and the acceptor intensity per pixel indicated no detectable intermolecular FRET.

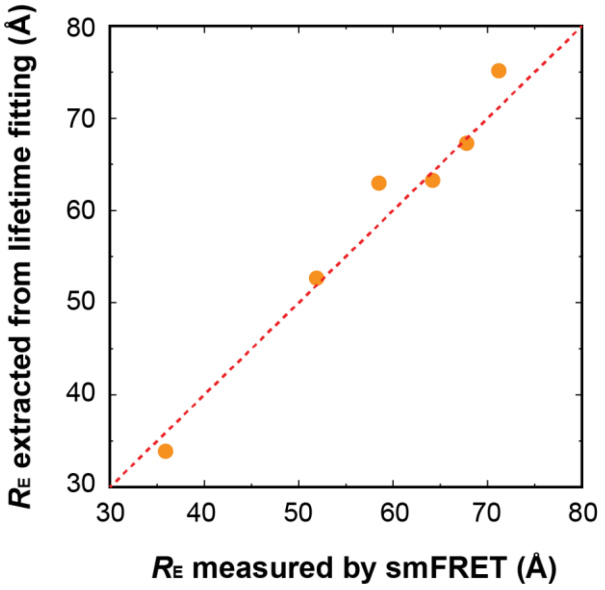

Supplementary Fig. 4 Correlation between the root-mean-square inter-residue distance R_E measured by intensity-based single-molecule FRET and R_E recovered from the lifetime-based fitting pipeline. In contrast to our in-cell measurements in main text Fig. 2, on the single-molecule level FRET efficiency can be measured as intensity-based or lifetime-based, and from both R_E can be computed independently. From the fluorescence measurements shown in Extended data Fig. 8, we fitted with Eqs. 11 and 12 and extracted the R_E for both strategies. The detected high correlation of $R^2 = 0.96$ validates the use of our FLIM-FRET pipeline to measure R_E. The red dotted line represents the reference line $y = x$.

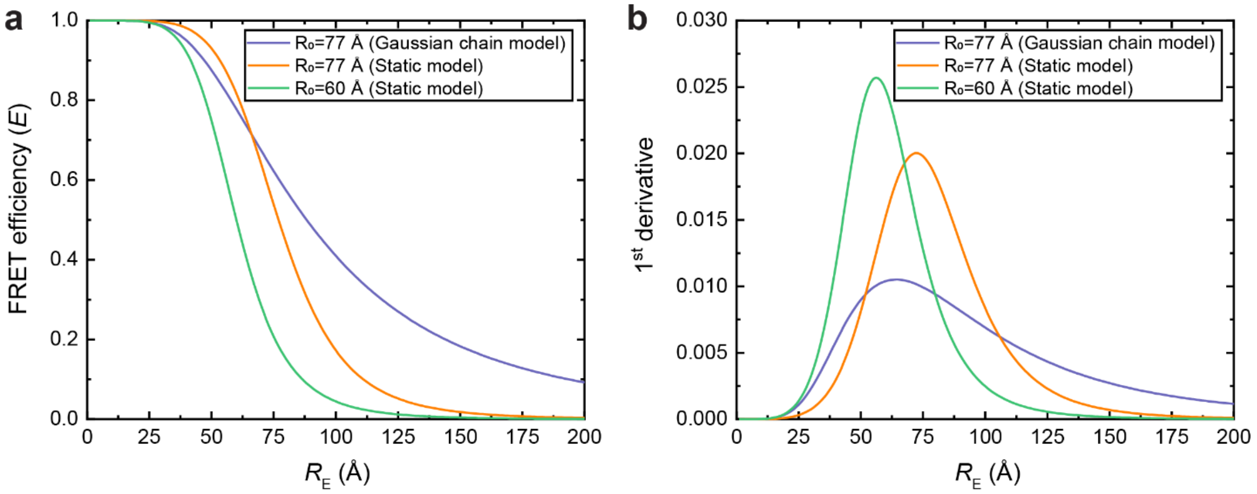

Supplementary Fig. 5 Comparison between a Gaussian chain model with a static model. (a) Theoretical FRET efficiency E as a function of root-mean-square inter-residue distance R_E for using dye pairs with different Förster distance R_0 and using different polymer models (Gaussian chain model vs Static model). **(b)** The 1st derivative of the plot in (a) can be used to estimate the sensitivity range for the FRET measurements, where the Gaussian chain model tails more towards larger distances.

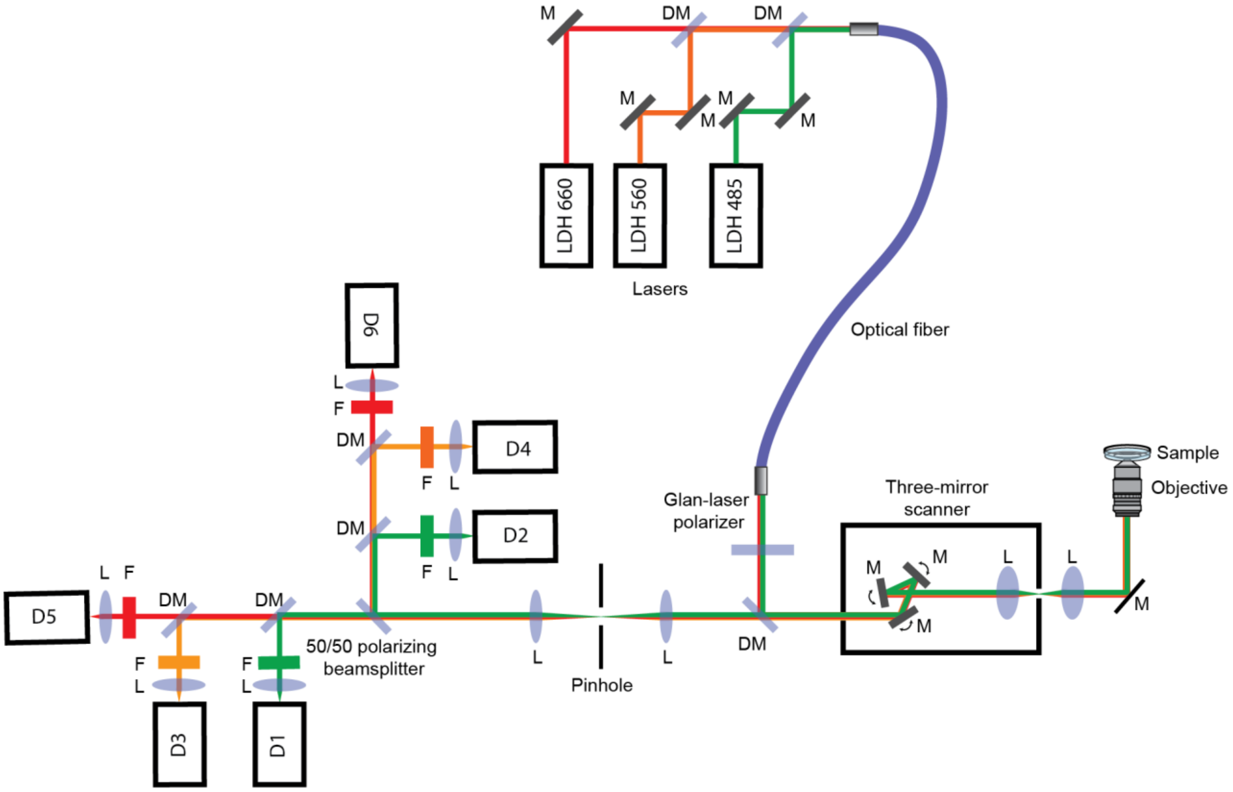

Supplementary Fig. 6 Schematic of the custom-built FLIM-FRET imaging system. Picosecond pulsed lasers diode heads including the wavelengths of 485 nm, 560 nm, and 660 nm were controlled through a multichannel picosecond diode laser driver. The beams were coupled into a single-mode polarization-maintaining optical fibre. The beam travelled through a Glan-laser polarizer and was directed into a laser scanning system. The three galvo mirrors in the scanning system were imaged onto the back focal plane of the objective with a 200 mm tube lens. The fluorescence emission was focused onto a pinhole, and then separated into parallel and perpendicular components using a 50/50 polarizing beam splitter. Each component was further separated by two sets of beam splitters, passed through three sets of bandpass filters, and focused onto the single-photon counting detectors. The signals from the photon detectors were recorded by a time-correlated single-photon counting (TCSPC) system. LDH, picosecond laser diode heads; L, lens; M, mirror; DM, dichroic mirror; D1, D3, D5, detectors perpendicular to the laser excitation; D2, D4, D6, detectors parallel to the laser excitation.

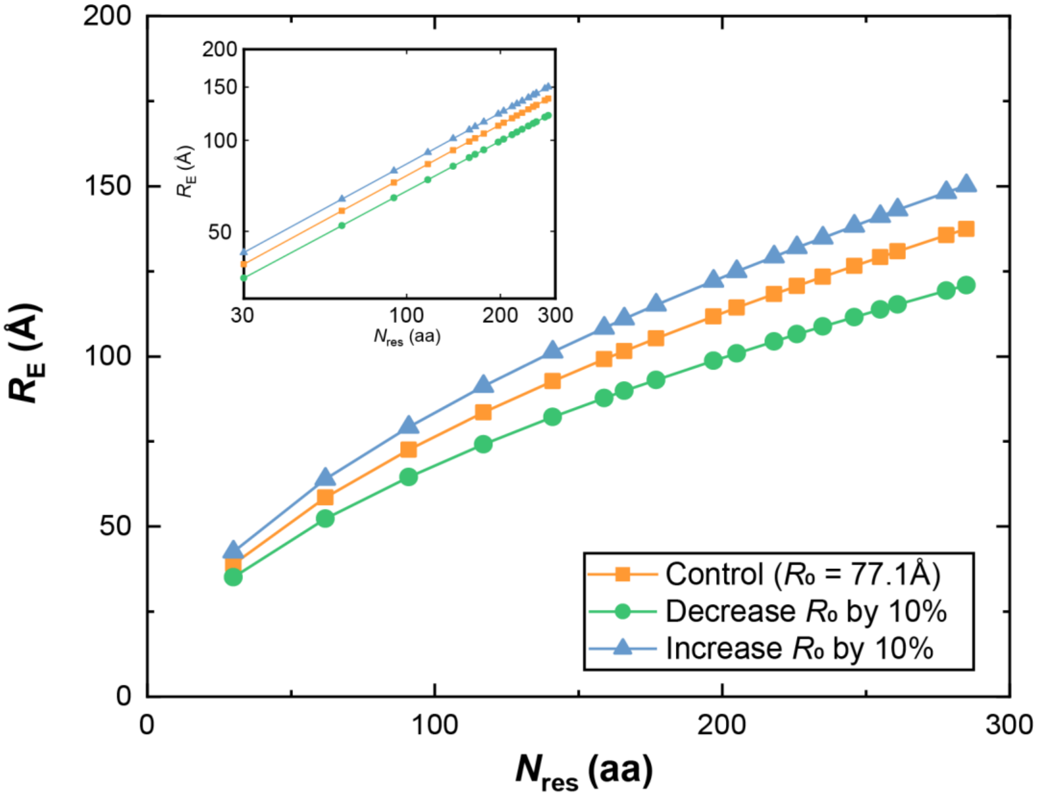

Supplementary Fig. 7 The apparent scaling exponent is robust to an error in Förster radius R_0 measurements. If the Förster radius R_0 increases or decreases by 10%, the apparent scaling exponent extracted from the global fitting of the lifetime decays for all mutants remains similar as (ν = 0.56, 0.56, and 0.55 for the cases with 10% increase, control, and 10% decrease, respectively), where only the prefactor of the scaling law changes (6.3, 5.8, and 5.4 for the cases with 10% increase, control, and 10% decrease, respectively).

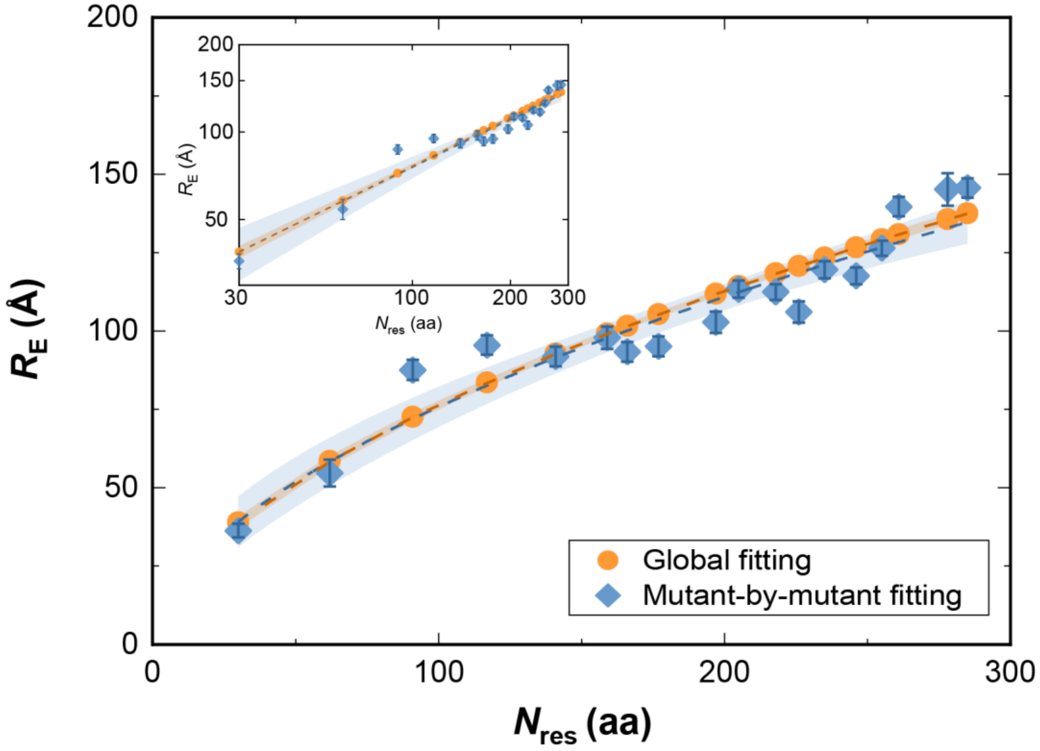

Supplementary Fig. 8 Comparison of the root-mean-square inter-residue distances R_E obtained from global fitting versus mutant-by-mutant fitting to the Gaussian chain model. Global fitting was performed by fitting the lifetime decays of all mutants with Eqs. 15, 16 and 18 to directly extract the scaling exponent over all approximately 2000 cells as well as the confidence interval from the bootstrap analysis (same as in Fig. 3 in the main text). Mutant-by-mutant fitting was performed by first extracting R_E for each mutant by fitting the lifetime decays of typically approximately 100 cells with Eqs. 15 and 16, and the error was estimated from the bootstrap analysis (indicated by the blue error bars). The scaling exponent was then obtained by fitting R_E versus N_{res} with Eq. 18. For the mutant-by-mutant fitting, the scaling exponent is $\nu = 0.55 \pm 0.05$, with the 95% confidence interval indicated by the blue error band.

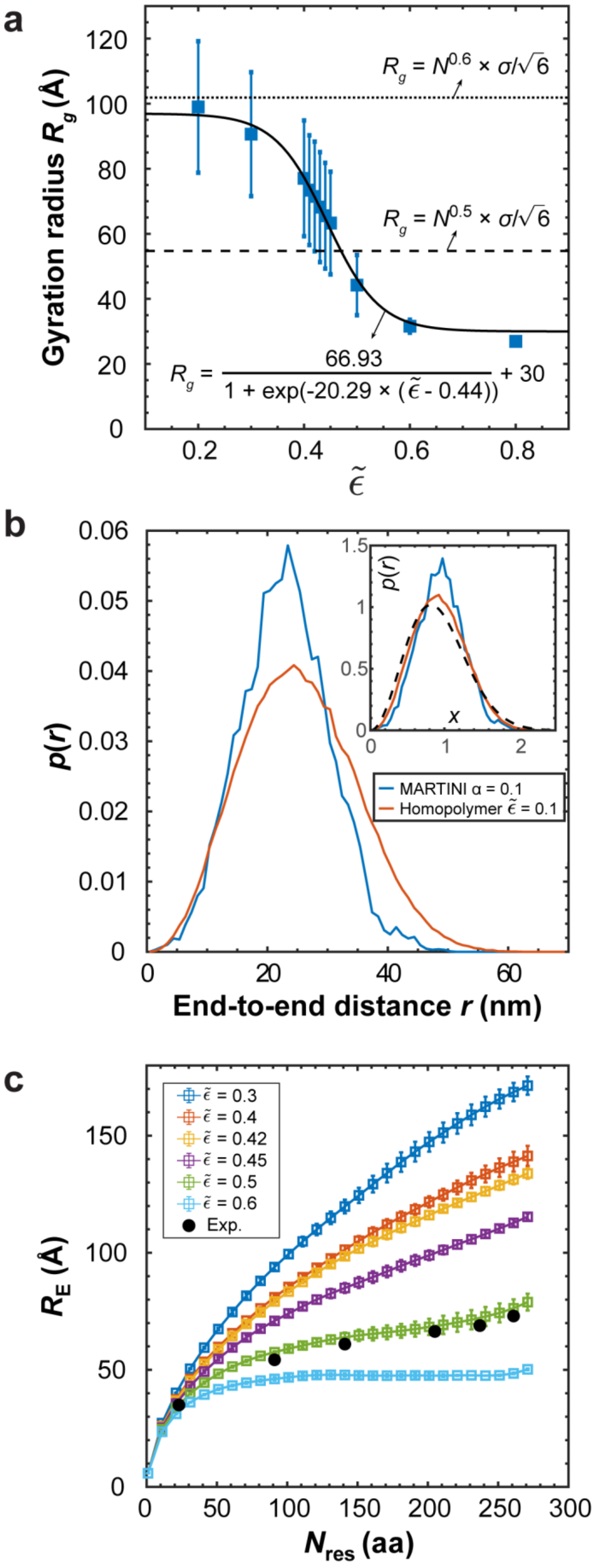

a
Gyration radius R_g (Å)
$R_g = N^{0.6} \times \sigma/\sqrt{6}$
$R_g = N^{0.5} \times \sigma/\sqrt{6}$
$R_g = \frac{66.93}{1 + \exp(-20.29 \times (\tilde{\epsilon} - 0.44))} + 30$
$\tilde{\epsilon}$
b
p(r)
End-to-end distance r (nm)
x
MARTINI α = 0.1
Homopolymer $\tilde{\epsilon}$ = 0.1
c
R_E (Å)
N_{res} (aa)
$\tilde{\epsilon}$ = 0.3
$\tilde{\epsilon}$ = 0.4
$\tilde{\epsilon}$ = 0.42
$\tilde{\epsilon}$ = 0.45
$\tilde{\epsilon}$ = 0.5
$\tilde{\epsilon}$ = 0.6
Exp.

Supplementary Fig. 9 Configuration of single NUP98 FG-chain (1-499) from MD simulations. (a) Radius of gyration of the homopolymer of size N = 499 as a function of cohesive strength $\tilde{\epsilon}$. The solid line shows a logistic function fitted to the data. The horizontal dotted and dashed lines indicate Flory's estimates for self-avoiding walks ($R_g = N^{0.6}\sigma/\sqrt{6} = 101.8$Å) and random coils ($R_g = N^{0.5}\sigma/\sqrt{6} = 54.7$Å), respectively. The error bar indicates the standard deviation of the chain's gyration radius calculated over the whole simulation time ($1.5 \times 10^6\tau$). The midpoint of the coil-globule transition is at $\tilde{\epsilon} = 0.44$. **(b)** Probability distribution of the end-to-end distance (r) for the homopolymer model ($\tilde{\epsilon} = 0.1$) and Martini model ($\alpha = 0.1$) of NUP98 FG-chain in the limit of weak cohesion. The inset shows the normalized end-to-end distance of the same data, $x = r/\langle r^2\rangle^{0.5}$, where $\langle r^2\rangle^{0.5}$ is the root-mean-square end-to-end distance R_{E}. The black dotted line is the Flory distribution function for a Gaussian chain, $p_{\mathrm{F}}(x) = 4\pi\left(\frac{3}{2\pi}\right)^{3/2} x^2 \exp(-1.5x^2)$, corresponding to Eq. 13 of the main text. **(c)** Root-mean-square inter-residue distance in single NUP98 FG-chain (1-499) from MD simulations versus residue separation. Solid black circles show the results of the smFRET measurements (from Extended Data Fig. 8). The symbols and error bars represent the average and standard error of the mean, respectively, as estimated from four blocks of size $4\times10^5\tau$.

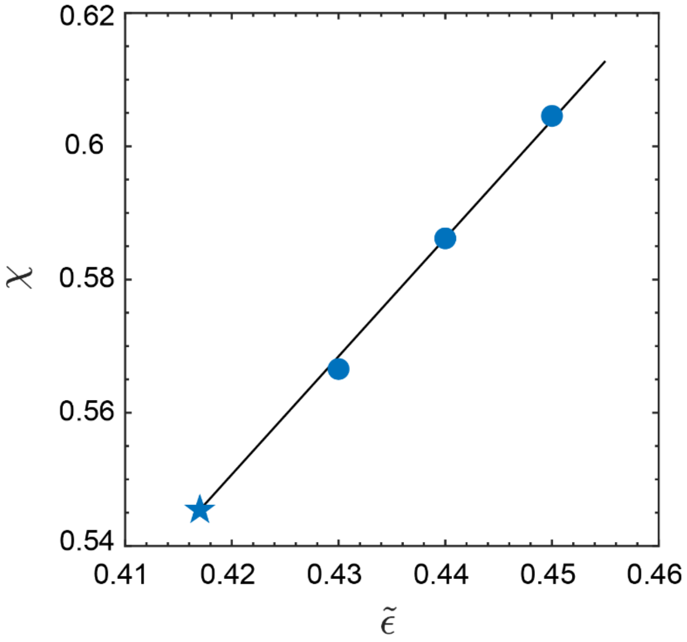

Supplementary Fig. 10 Linear relation between the Flory parameter χ from Eq. 22 and the cohesive strength $\tilde{\epsilon}$ of NUP98-FG (1-499). The line shows a linear fit with slope $A = 1.773$ and intercept $B = -0.1938$. The blue star indicates the critical interaction strength at $\chi_c = 0.545$ and $\tilde{\epsilon}_c = 0.417$.

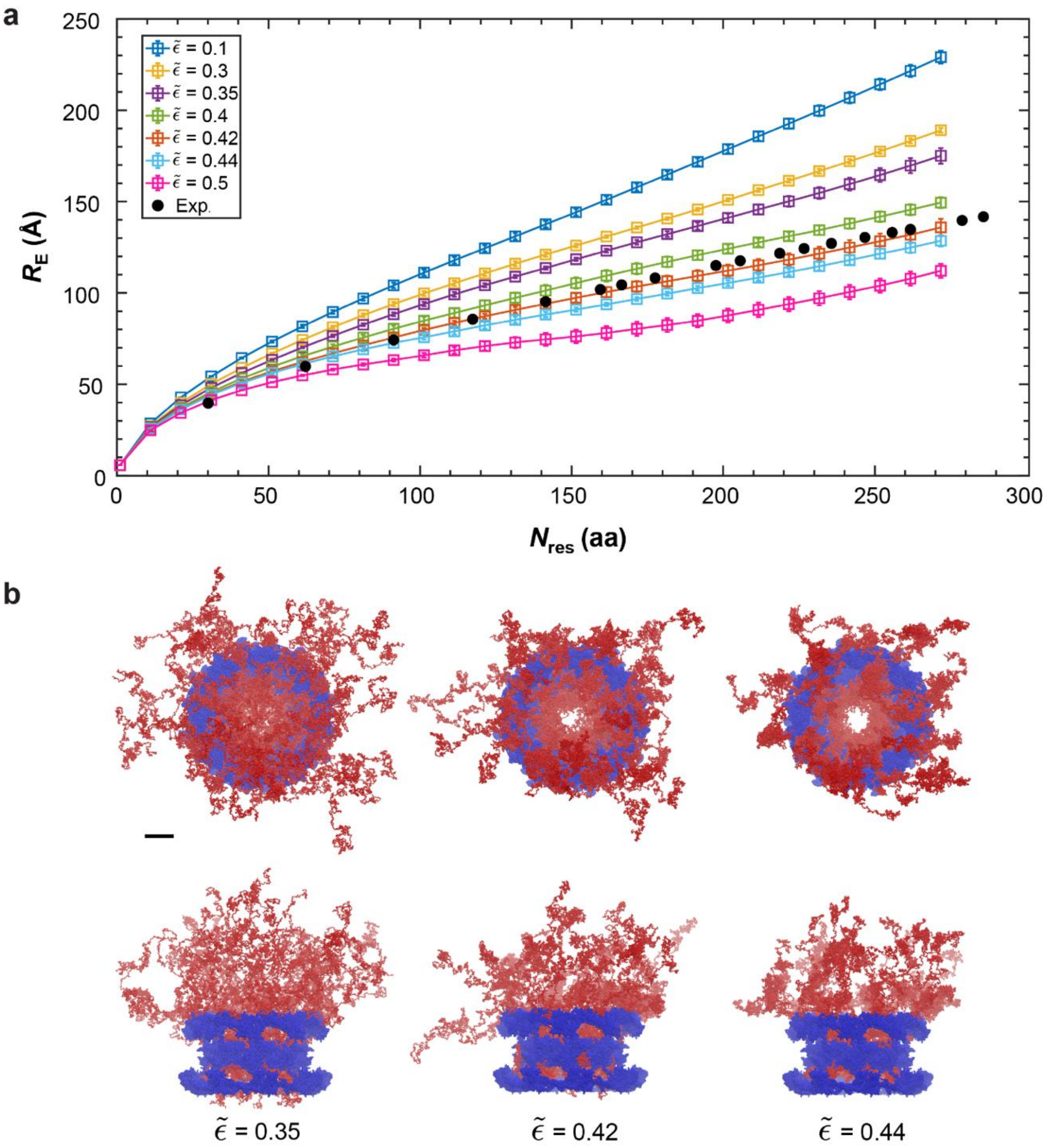

Supplementary Fig. 11 Coarse-grained MD simulations of FG-NUPs in NPC model I (see Supplementary Table 2). (**a**) Root-mean-square inter-residue distance R_{E} of beads on the same NUP98 FG domain as function of residue separation N_{res}. The different effective NUP-NUP interaction strengths $\tilde{\epsilon}$ are indicated in the legend. The distances from the FLIM-FRET experiments are shown as filled black circles (Fig. 3 in the main text), which superimpose on the simulation data for $\tilde{\epsilon} = 0.42$. The symbols and error bars represent the average and standard error of the mean, respectively, as estimated from four blocks of size $10^4\tau$. (**b**) Side and top views of the NPC at the end of the MD simulations with $\tilde{\epsilon} = 0.35$, $\tilde{\epsilon} = 0.42$, and $\tilde{\epsilon} = 0.44$ (left to right; scaffold: blue; FG-NUPs: red, scale bar: 20 nm).

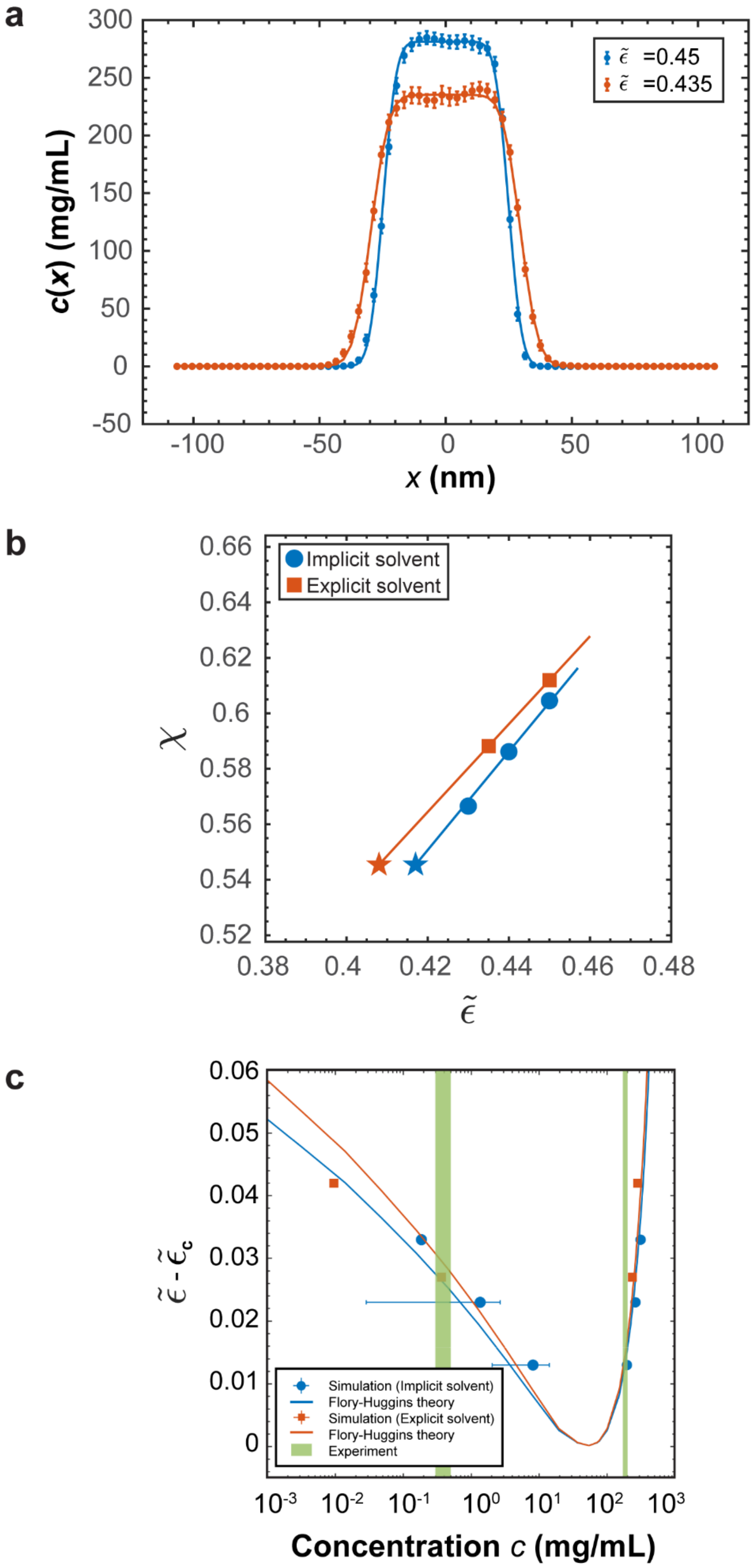
a
c(x) (mg/mL)
x (nm)
ε̃ =0.45
ε̃ =0.435
b
Implicit solvent
Explicit solvent
χ
ε̃
c
ε̃ - ε̃c
Concentration c (mg/mL)
Simulation (Implicit solvent)
Flory-Huggins theory
Simulation (Explicit solvent)
Flory-Huggins theory
Experiment

Supplementary Fig. 12 Phase diagram of NUP98 FG condensate with explicit solvent. (a) Concentration profiles along the long box direction in MD simulations of 50 NUP98(1-499) FG chains in explicit solvent. The cohesive interaction strengths $\tilde{\epsilon}$ between the monomers are indicated in the legend. The symbols and error bars represent the average and standard deviation of time-averaged density profiles over $3\times10^4\tau$. **(b)** Linear relations between the Flory parameter (χ) and the cohesive interaction strength ($\tilde{\epsilon}$) for simulations in implicit solvent (blue) and explicit solvent (red). The critical cohesive values are shown with stars and obtained as $\tilde{\epsilon}_c \approx 0.417$ for implicit solvent and $\tilde{\epsilon}_c \approx 0.41$ for explicit solvent, respectively. Lines are fits to the simulation data (squares and circles; see legend). **(c)** Phase diagram of the NUP98 FG-condensate in the plane cohesive interaction strength and concentrations c of coexisting phases. The coexistence curves obtained for implicit solvent (blue) and explicit solvent (red) are shifted vertically by the respective critical cohesive strength, $\tilde{\epsilon} - \tilde{\epsilon}_c$. The solid blue and red lines are the result of Flory-Huggins theory for a homopolymer of a length N = 499. For $\tilde{\epsilon} = 0.435$ and $\tilde{\epsilon} = 0.45$, no chain escaped the condensate during the MD simulations. As in Extended Data Fig. 10c, we estimated the dilute-phase density by fitting a double error function to the averaged concentration profile. For $\tilde{\epsilon} = 0.435$, we obtained $c_{dense} = 235.21$ mg/mL, $c_{dilute} = 0.37$ mg/mL, $B = 29.63$nm and $w = 7.81$nm. For $\tilde{\epsilon} = 0.45$, we obtained $c_{dense} = 281.2$ mg/mL, $c_{dilute} = 0.01$ mg/mL, $B = 24.88$ nm and $w = 5.92$ nm. Reported concentrations of the dense and dilute phases are indicated as green bars[27,54].

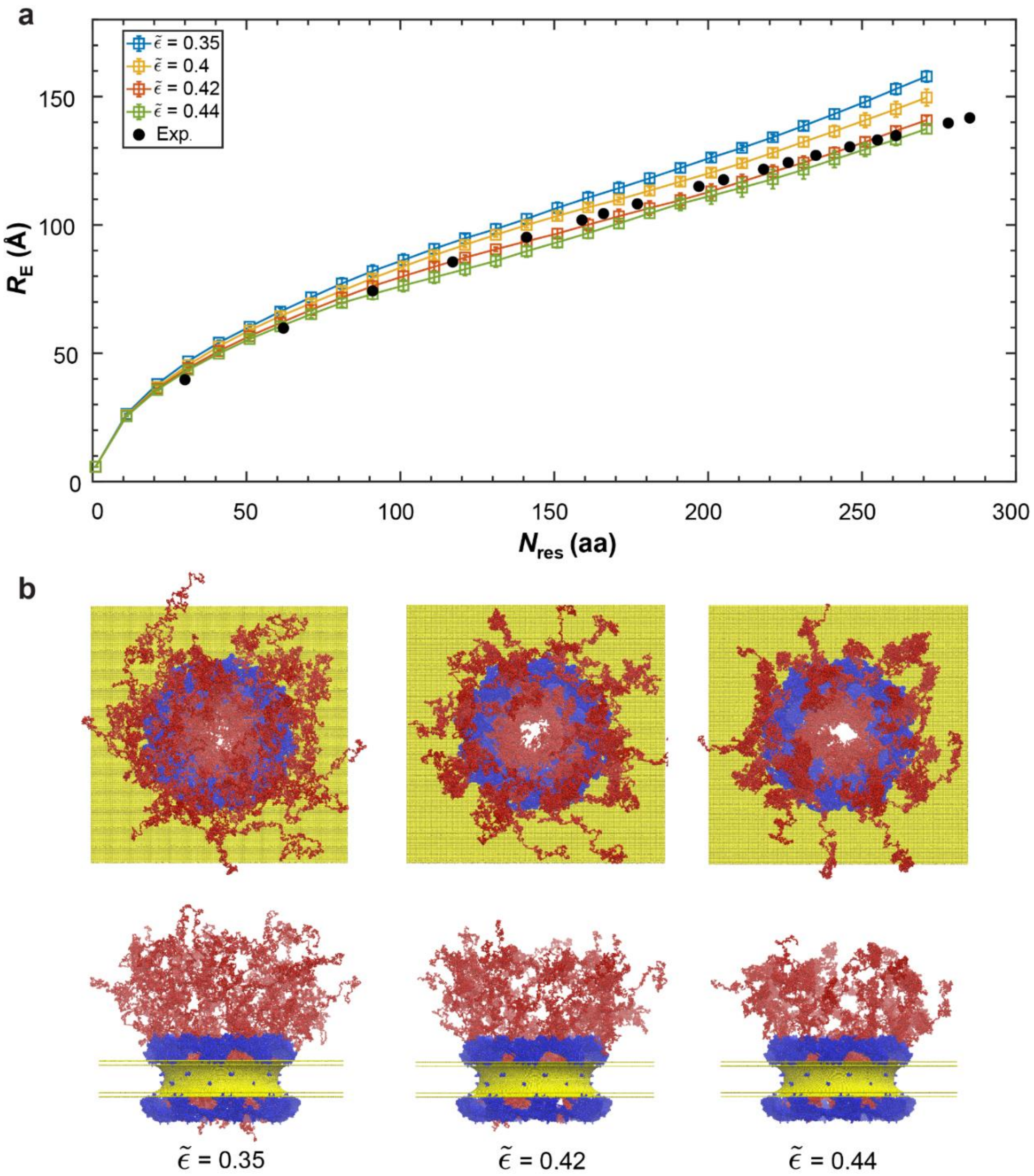

Supplementary Fig. 13 Coarse-grained MD simulations of FG-NUPs in NPC model I with explicit solvent (Supplementary Table 4). (**a**) Root-mean-square inter-residue distance R_E of beads on the same NUP98 FG domain in the NPC as function of residue separation N_{res} for different effective NUP-NUP interaction strengths $\tilde{\epsilon}$ (see legend). The distances from the FLIM-FRET experiments are shown as filled circles (from Fig. 3 in the main text). The symbols and error bars represent the average and standard error of the mean, respectively, as estimated from four blocks of size $4\times10^3\tau$. (**b**) Side and top views of the NPC at the end of the MD simulations with $\tilde{\epsilon} = 0.35$, $\tilde{\epsilon} = 0.42$, and $\tilde{\epsilon} = 0.44$ (left to right; scaffold: blue; membrane envelope: yellow; FG-NUPs: red, scale bar: 20 nm).

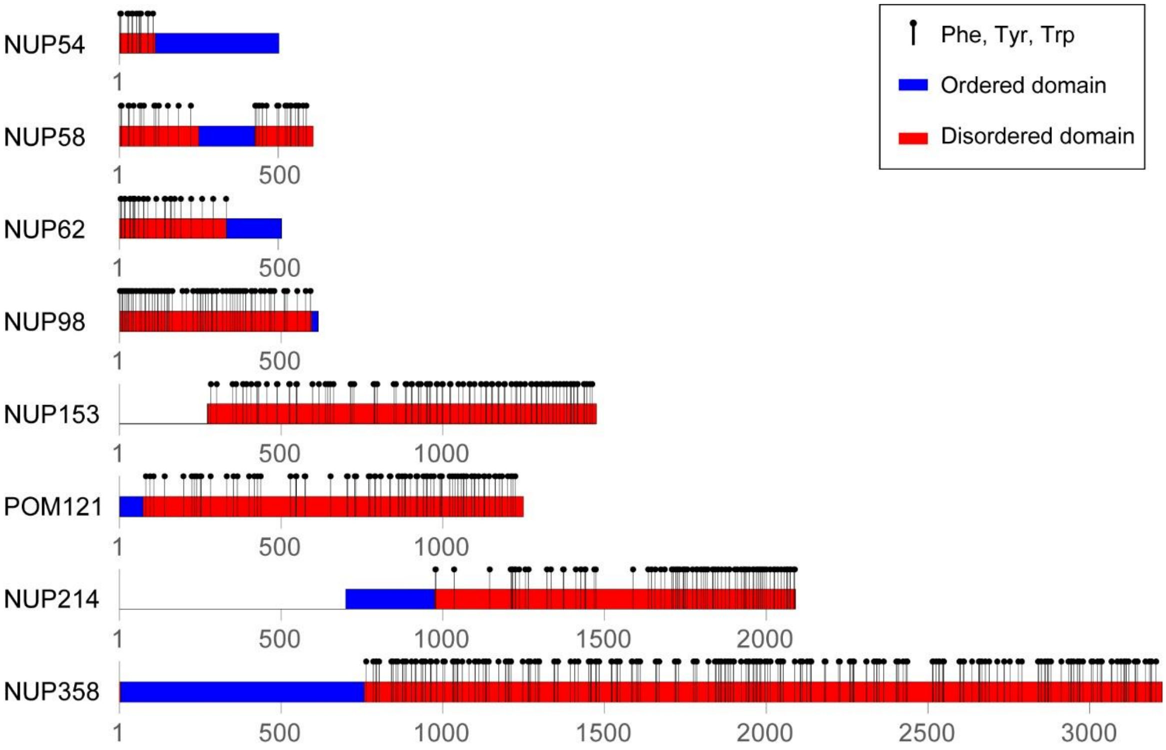

Supplementary Fig. 14 Distribution of aromatic residues along the FG-NUPs in FYW-stickers model of NPC. Black stems indicate the positions of aromatic residues (Phe, Tyr, Trp) FG-NUP sequences modelled as disordered domains (red). Ordered domains are indicated as blue stripes.

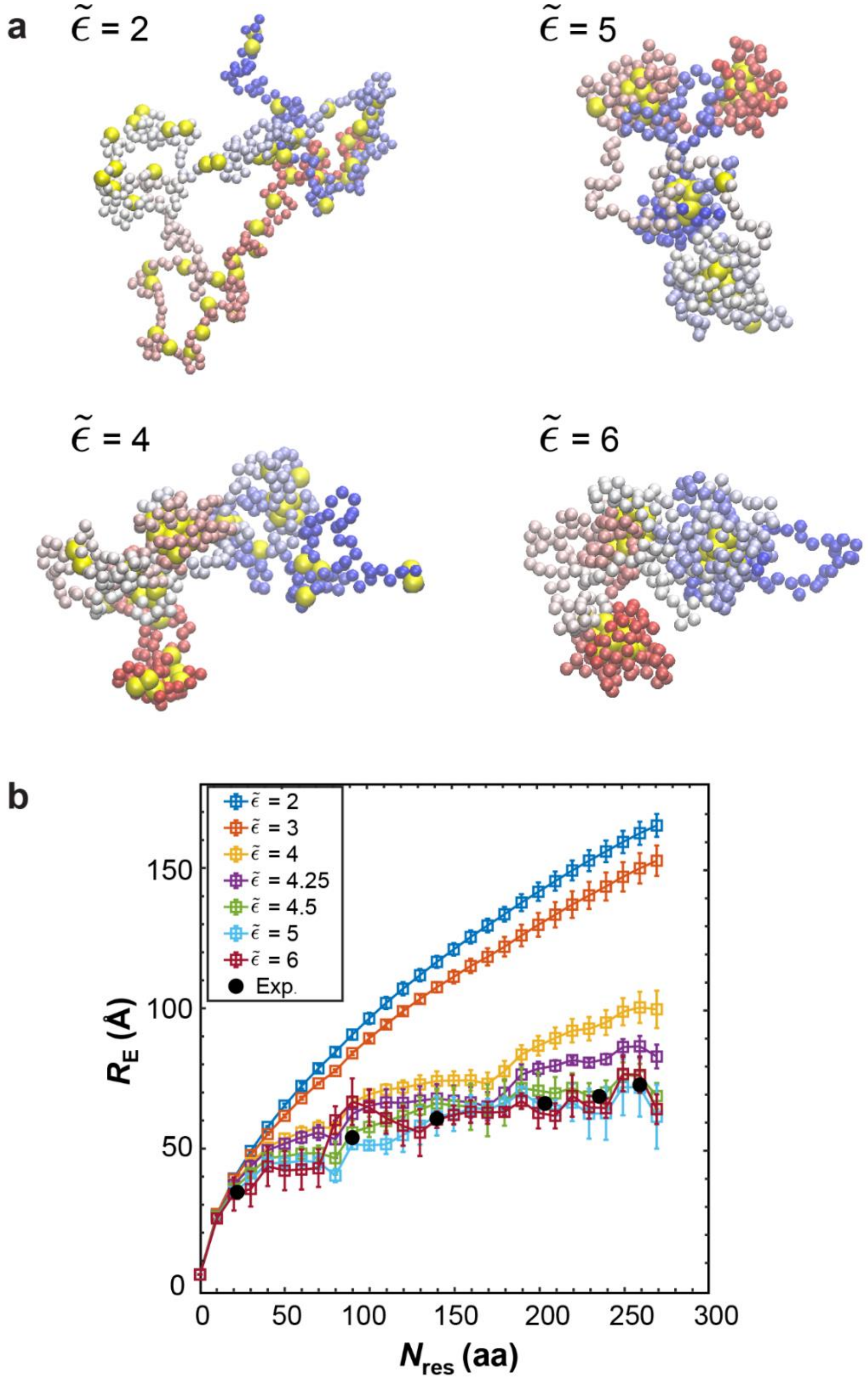

Supplementary Fig. 15 Single-chain conformations of FYW-stickers model of NUP98. (**a**) Conformations of a single NUP98(1-499) chain for different interaction strengths between the stickers. The chain beads are coloured by index number (N-to-C: blue-white-red). Phe, Trp, and Tyr residues are coloured in yellow. (**b**) Root-mean-square inter-residue distance R_E in single NUP98(1-499) chain from MD simulations with FYW-stickers model versus residue separation N_{res}. Filled black circles show the results of the smFRET measurements (from Extended Data Fig. 8). The symbols and error bars represent the average and standard error of the mean, respectively, as estimated from four blocks of size $8{\times}10^4\tau$.

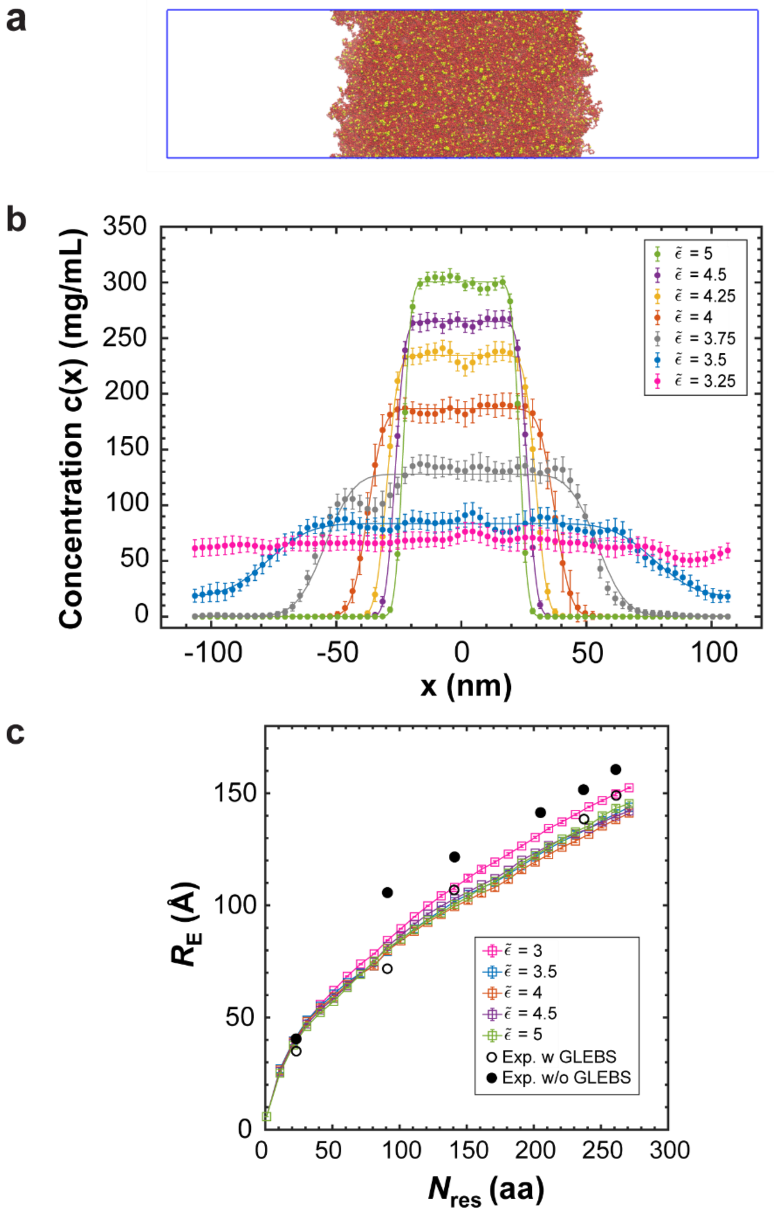

Supplementary Fig. 16 Condensate propensity of FYW-stickers model of NUP98. (**a**) Side-view of MD simulation snapshot showing a condensate of 500 NUP98 chains described by the FYW-stickers model with sticker interaction strength $\tilde{\epsilon} = 4$. Aromatic residues (F, Y, W) are colored in yellow and all other residues in red. (**b**) Concentration profiles of the condensate for different interaction strengths between the stickers along the long axis of the elongated simulation box. The symbols and error bars represent the average and standard deviation, respectively, of time-averaged density profiles over $2.4\times10^5\tau$. (**c**) Root-mean-square inter-residue distance R_E in single NUP98(1-499) chain in condensate from MD simulations with FYW-stickers model versus residue separation N_{res}. Empty and filled black circles show the results of the FLIM-FRET measurements (from Extended Data Fig. 10d). The symbols and error bars represent the average and standard error of the mean, respectively, as estimated from four blocks of size $8\times10^4\tau$.

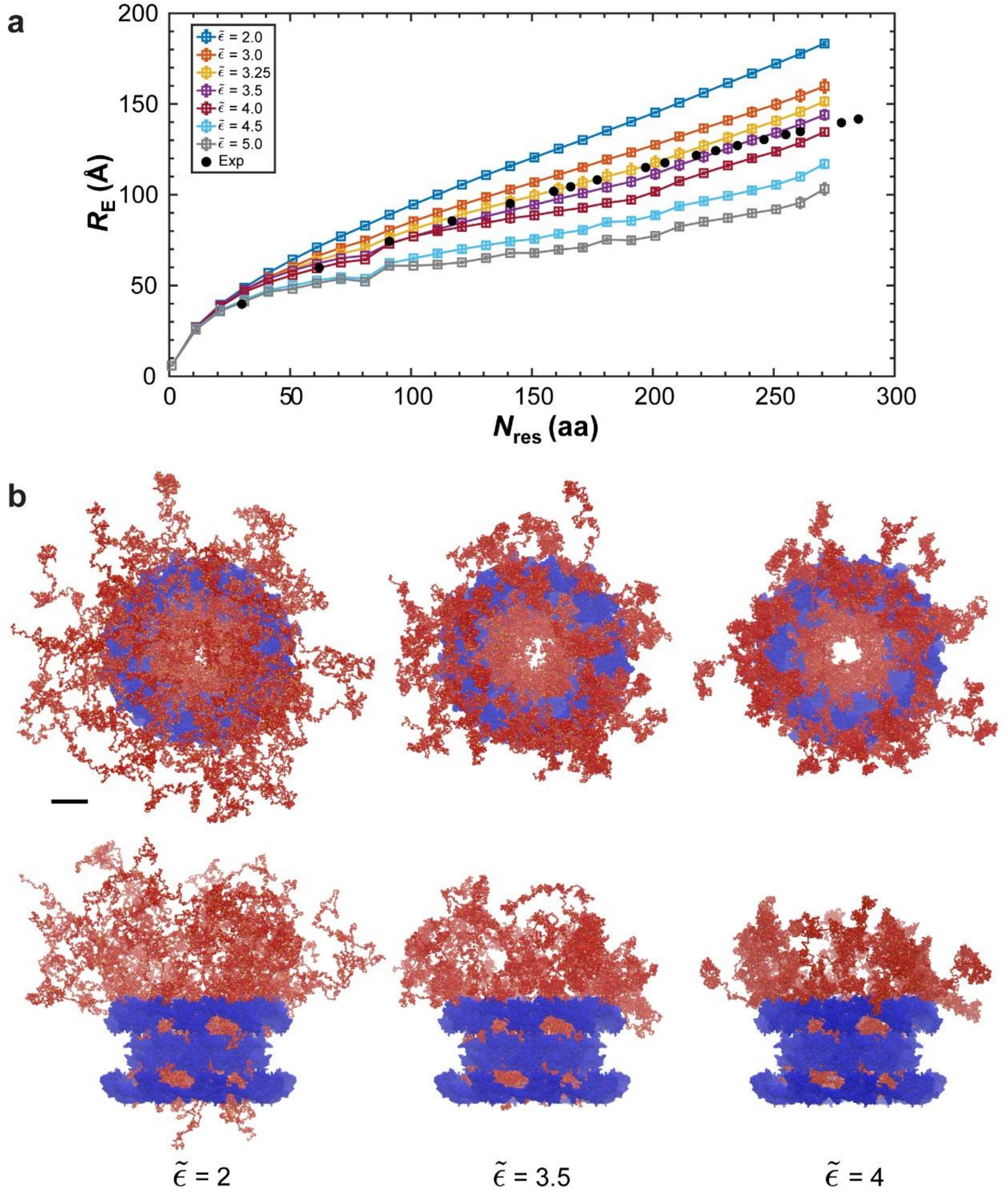

Supplementary Fig. 17 Coarse-grained MD simulations of NPC with FYW-stickers model. (**a**) Root-mean-square inter-residue distance R_{E} of beads on the same NUP98 FG domain in the NPC as function of residue separation N_{res} with different effective NUP-NUP interaction strength $\tilde{\epsilon}$, as indicated in the legend. The distances from the FLIM-FRET experiments are shown as filled black circles (from Fig. 3 in the main text). The symbols and error bars represent the average and standard error of the mean, respectively, as estimated from four blocks of size $4{\times}10^3\tau$. (**b**) Side and top views of the NPC at the end of the MD simulations with $\tilde{\epsilon} = 2$, $\tilde{\epsilon} = 3.5$, and $\tilde{\epsilon} = 4$ (left to right; scaffold: blue; Phe, Trp, and Tyr residues: yellow; other residues of FG-NUPs: red, scale bar: 20 nm).

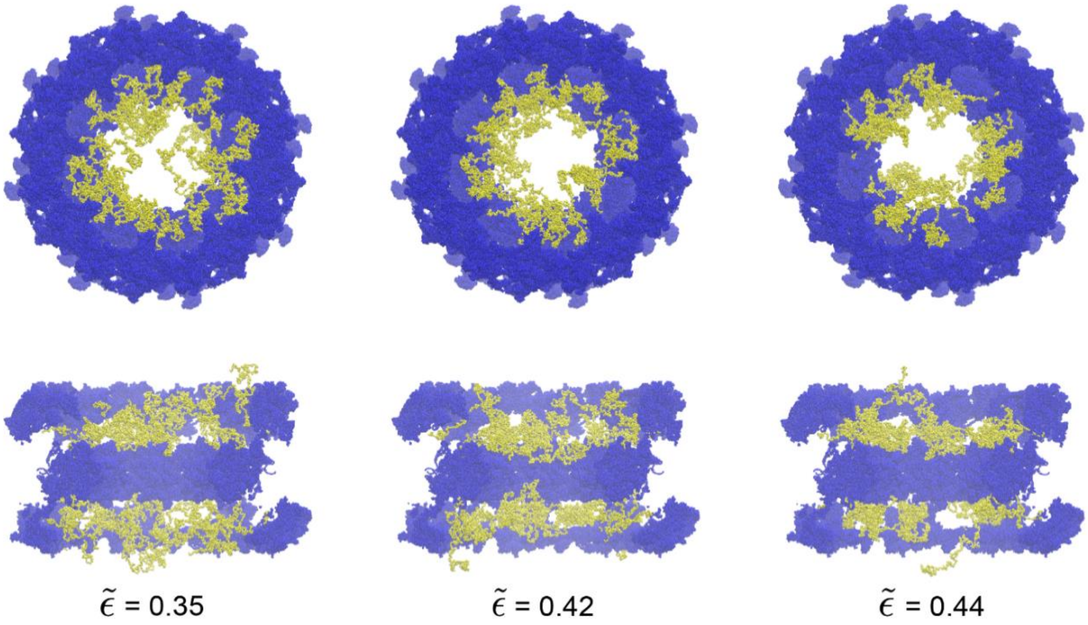

Supplementary Fig. 18 Conformations of NUP98 inside NPC. Side and top views of the NUP98 chains inside NPC with $\tilde{\epsilon} = 0.35$, $\tilde{\epsilon} = 0.42$, and $\tilde{\epsilon} = 0.44$ (left to right) in MD simulations with NPC model II (see Supplementary Table 3). The other FG-NUPs, including NUP98, are shown in Fig. 4b. The side views are cut at the NPC centre (scaffold: blue; NUP98 chains: yellow).

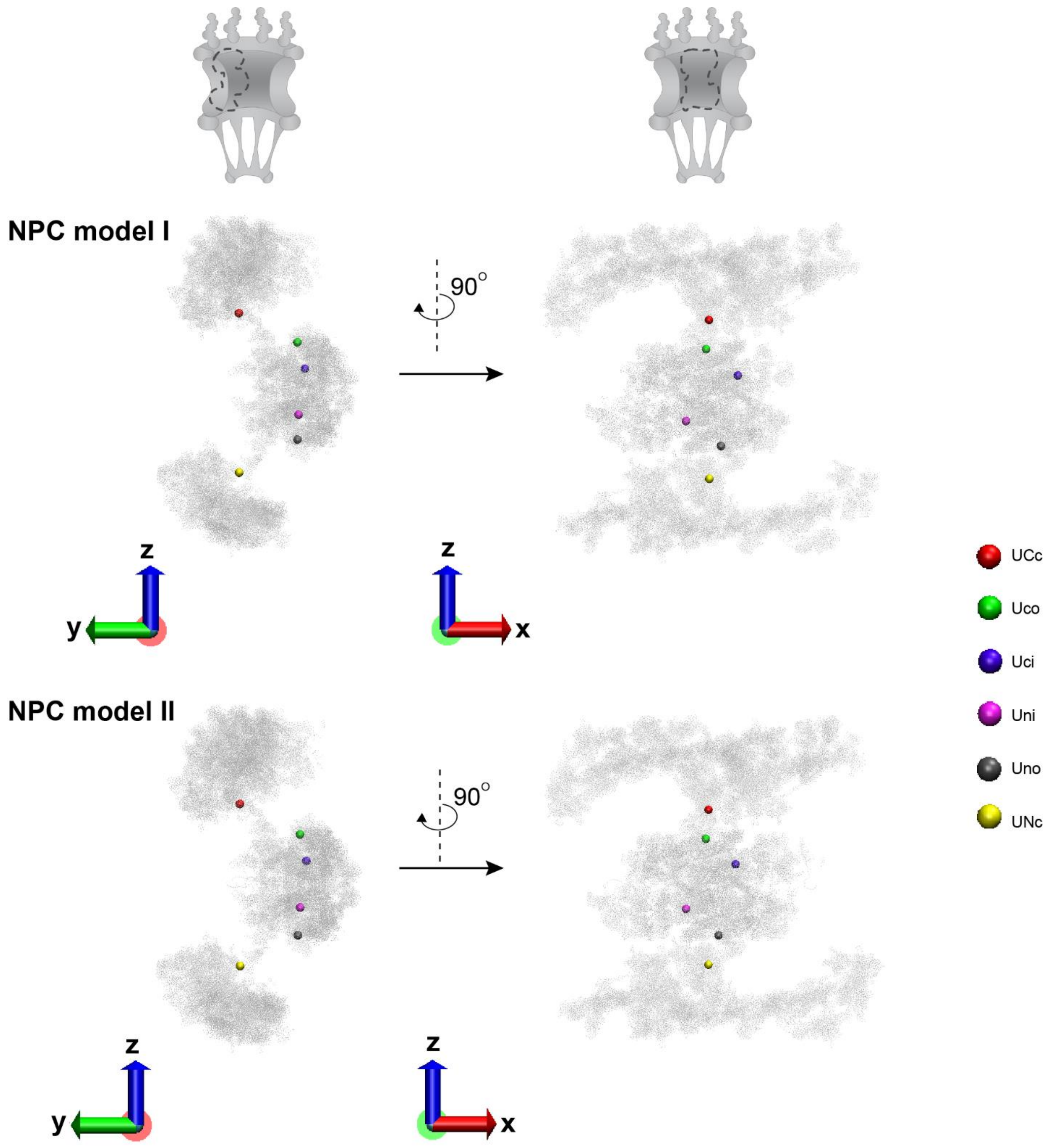

Supplementary Fig. 19 Grafting sites of NUP98 inside the NPC. Arc and radial views of the NUP98 grafting positions on one symmetry subunit of NPC model I (top row, see Supplementary Table 2) and NPC model II (bottom row, see Supplementary Table 3). NPC scaffold residues are shown as silver points. Coordinates for NPC models I and II are provided as Supplementary Data 1 and 2. Visualization using VMD[85]. The grafting sites for NUP98 are based on the short fragments of the NUP98 that are bound to the NUP155. There are 6 copies of NUP155 per spoke in the human NPC, and we call them: cytoplasmic outer (co), cytoplasmic inner (ci), nuclear outer (no), nuclear inner (ni), and two so-called NUP155 connectors, one on the cytoplasmic side (Cc), one

on the nuclear side (Nc). Accordingly, we named the grafting sites of the NUP98 as: UCc - NUP98 bound to the NUP155 connector on the cytoplasmic side; Uco - NUP98 bound to the NUP155 cytoplasmic outer copy; Uci - NUP98 bound to the NUP155 cytoplasmic outer copy; Uni - NUP98 bound to the NUP155 nuclear inner copy; Uno - NUP98 bound to the NUP155 nuclear outer copy; UNc - NUP98 bound to the NUP155 connector on the nuclear side.

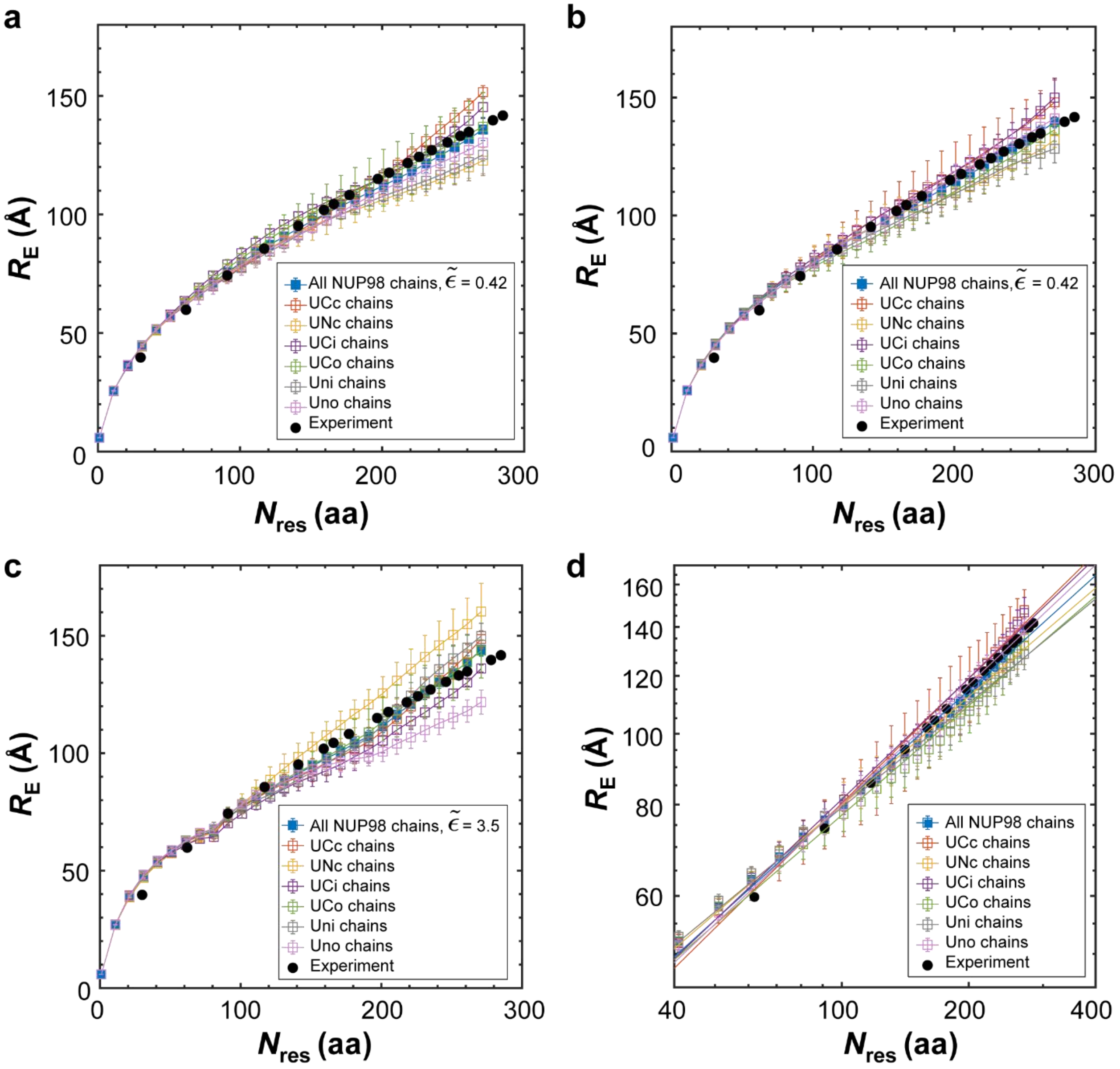

Supplementary Fig. 20 Root-mean-square inter-residue distance R_E of NUP98 FG domains grouped by the grafting positions on the NPC scaffold in the MD simulations. (a-c) R_E of beads on NUP98 FG domain in the NPC as function of residue separation N_{res} for homopolymer model I (**a**), homopolymer model II (**b**), and FYW stickers model (**c**). The 8-fold symmetric human NPC contains 8x6=48 NUP98 chains with six distinct anchoring positions (see Supplementary Tables 2 and 3, and Supplementary Fig. 19). The symbols connected by lines in each panel show the averaged R_E for each position. The effective NUP-NUP interaction strengths are $\tilde{\epsilon} = 0.42$ for homopolymer models I and II, and $\tilde{\epsilon} = 3.5$ for the FYW-stickers model. (**d**) Distance scaling of NUP98 FG domains grouped by the grafting positions in NPC model II on a log-log scale for an interaction strength of $\tilde{\epsilon} = 0.42$. Lines show fits to a scaling law, $R_E = aN_{res}^{\nu}$, with parameters a and ν. The fitting parameter values are $a = 5.88$ Å and $\nu = 0.567$ for UCc chains; $a = 8.23$ Å and $\nu = 0.493$ for UNc chains; $a = 6.5$ Å and $\nu = 0.549$ for Uci chains; $a = 7.84$ Å and $\nu =$

0.497 for Uco chains; $a = 8.94$ Å and $\nu = 0.474$ for Uni chains; $a = 6.53$ Å and $\nu = 0.544$ for Uno chains and $a = 7.2$ Å and $\nu = 0.520$ for all NUP98 chains. The average and standard deviation of scaling exponents in the MD simulations are $\nu_{sim} = 0.521 \pm 0.038$. For reference, the experimental distances are shown as black circles ($\nu = 0.56 \pm 0.03$, same dataset as in Fig. 3). For (a-d), the symbols and error bars represent the average and standard error of the mean, respectively, as estimated from four blocks of size $10^4\tau$.

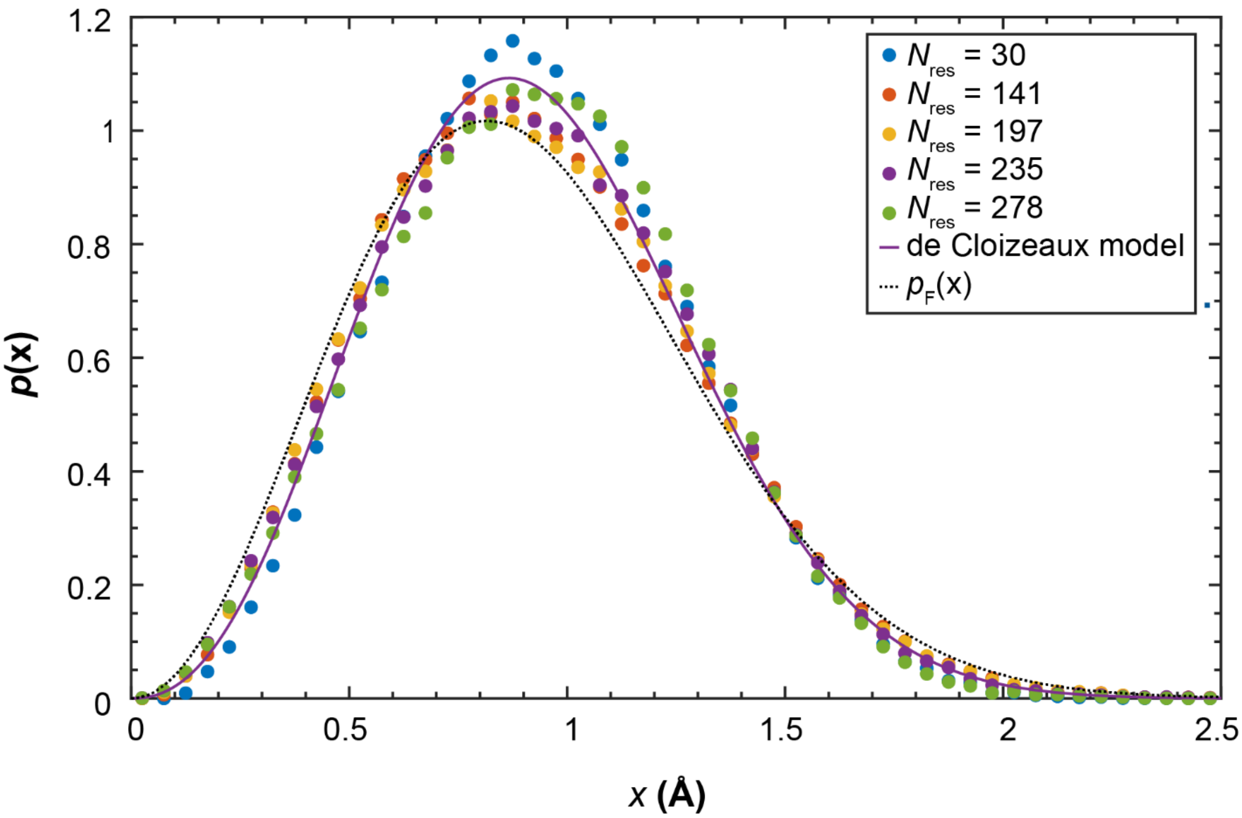

Supplementary Fig. 21 Normalized inter-residue distance distribution functions for NUP98 chains inside NPC model II. Symbols show distributions of normalized distances, $x = r/\langle r^2 \rangle^{0.5}$, between residues 221 and 221+N_{res} (see legend), where r and $\langle r^2 \rangle^{0.5}$ are the distance and root-mean-square inter-residue distance, respectively. The distributions were averaged over all NUP98 chains at interaction strength $\tilde{\epsilon} = 0.42$. By construction, the distributions satisfy $\int_0^\infty p(x)dx = 1$ and $\int_0^\infty p(x)x^2 dx = 1$. For reference, the violet solid line is the de Cloizeaux distribution Eq. 19 underlying the SAW-ν model of Zheng et al.[48] for a self-avoiding random walk with $\nu = 0.56$, $\delta = 1/(1-\nu) \approx 2.273$, $g = (\gamma - 1)/\nu \approx 0.29$ for $\gamma = 1.1615$ and after appropriate transformation to the normalized distance x. The black dotted line is the Flory distribution function for a Gaussian chain, $p_{\mathrm{F}}(x) = 4\pi \left(\frac{3}{2\pi}\right)^{3/2} x^2 \exp(-1.5x^2)$, corresponding to Eq. 13 in the main text.

Supplementary Table 1. Plasmids used in this work

Construct	In Figure
pcDNA3.1-FLAG-hsNUP98^{A221TAG}-boxB	Fig.1-3, Extended data Fig.1-5
pcDNA3.1-FLAG-hsNUP98$^{A221TAG-A251TAG}$-boxB	Fig.2-3, Extended data Fig.5-6, Supplementary Fig. 3
pcDNA3.1-FLAG-hsNUP98$^{A221TAG-S283TAG}$-boxB	Fig.2-3, Extended data Fig.5, Supplementary Fig. 3
pcDNA3.1-FLAG-hsNUP98$^{A221TAG-S312TAG}$-boxB	Fig.2-3, Extended data Fig.5, Supplementary Fig. 3
pcDNA3.1-FLAG-hsNUP98$^{A221TAG-S338TAG}$-boxB	Fig.2-3, Extended data Fig.5, Supplementary Fig. 3
pcDNA3.1-FLAG-hsNUP98$^{A221TAG-S362TAG}$-boxB	Fig.2-3, Extended data Fig.5, Supplementary Fig. 3
pcDNA3.1-FLAG-hsNUP98$^{A221TAG-A380TAG}$-boxB	Fig.2-3, Extended data Fig.5, Supplementary Fig. 3
pcDNA3.1-FLAG-hsNUP98$^{A221TAG-S387TAG}$-boxB	Fig.2-3, Extended data Fig.5, Supplementary Fig. 3
pcDNA3.1-FLAG-hsNUP98$^{A221TAG-S398TAG}$-boxB	Fig.2-3, Extended data Fig.5, Supplementary Fig. 3
pcDNA3.1-FLAG-hsNUP98$^{A221TAG-A418TAG}$-boxB	Fig.2-3, Extended data Fig.5, Supplementary Fig. 3
pcDNA3.1-FLAG-hsNUP98$^{A221TAG-A426TAG}$-boxB	Fig.2-3, Extended data Fig.5, Supplementary Fig. 3
pcDNA3.1-FLAG-hsNUP98$^{A221TAG-I439TAG}$-boxB	Fig.2-3, Extended data Fig.5, Supplementary Fig. 3
pcDNA3.1-FLAG-hsNUP98$^{A221TAG-A447TAG}$-boxB	Fig.2-3, Extended data Fig.5, Supplementary Fig. 3
pcDNA3.1-FLAG-hsNUP98$^{A221TAG-A458TAG}$-boxB	Fig.2-3, Extended data Fig.5, Supplementary Fig. 3
pcDNA3.1-FLAG-hsNUP98$^{A221TAG-A467TAG}$-boxB	Fig.2-3, Extended data Fig.5, Supplementary Fig. 3
pcDNA3.1-FLAG-hsNUP98$^{A221TAG-A476TAG}$-boxB	Fig.2-3, Extended data Fig.5, Supplementary Fig. 3
pcDNA3.1-FLAG-hsNUP98$^{A221TAG-A482TAG}$-boxB	Fig.2-3, Extended data Fig.5-6, Supplementary Fig. 3
pcDNA3.1-FLAG-hsNUP98$^{A221TAG-S499TAG}$-boxB	Fig.2-3, Extended data Fig.5, Supplementary Fig. 3
pcDNA3.1-FLAG-hsNUP98$^{A221TAG-M506TAG}$-boxB	Fig.2-3, Extended data Fig.5, Supplementary Fig. 3
pcDNA3.1-TOM20$_{1-70}$-FUS$_{1-478}$-4xλ_{N22}-PylRS^{Y306A,Y384F}-U6-tRNA^{Pyl}	Fig.1-3, Extended data Fig.1-6, Supplementary Fig. 3
pcDNA3.1-NES- PylRS^{Y306A,Y384F}-U6-tRNA^{Pyl}	Extended data Fig.1
pcDNA3.1-FLAG-hsNUP98^{A251TAG}-boxB	Extended data Fig.4

pcDNA3.1-FLAG-hsNUP98^{S283TAG}-boxB	Extended data Fig.4
pcDNA3.1-FLAG-hsNUP98^{S312TAG}-boxB	Extended data Fig.4
pcDNA3.1-FLAG-hsNUP98^{S338TAG}-boxB	Extended data Fig.4
pcDNA3.1-FLAG-hsNUP98^{S362TAG}-boxB	Extended data Fig.4
pcDNA3.1-FLAG-hsNUP98^{A380TAG}-boxB	Extended data Fig.4
pcDNA3.1-FLAG-hsNUP98^{S387TAG}-boxB	Extended data Fig.4
pcDNA3.1-FLAG-hsNUP98^{S398TAG}-boxB	Extended data Fig.4
pcDNA3.1-FLAG-hsNUP98^{A418TAG}-boxB	Extended data Fig.4
pcDNA3.1-FLAG-hsNUP98^{A426TAG}-boxB	Extended data Fig.4
pcDNA3.1-FLAG-hsNUP98^{I439TAG}-boxB	Extended data Fig.4
pcDNA3.1-FLAG-hsNUP98^{A447TAG}-boxB	Extended data Fig.4
pcDNA3.1-FLAG-hsNUP98^{A458TAG}-boxB	Extended data Fig.4
pcDNA3.1-FLAG-hsNUP98^{A467TAG}-boxB	Extended data Fig.4
pcDNA3.1-FLAG-hsNUP98^{A476TAG}-boxB	Extended data Fig.4
pcDNA3.1-FLAG-hsNUP98^{A482TAG}-boxB	Extended data Fig.4
pcDNA3.1-FLAG-hsNUP98^{S499TAG}-boxB	Extended data Fig.4
pcDNA3.1-FLAG-hsNUP98^{M506TAG}-boxB	Extended data Fig.4
pQE-14His-TEV-hsNUP98$_{FG\ 1\text{-}505,\ \Delta GLEBS}$	Fig.1 and 3, Extended data Fig.3, 9 and 10
pQE-14His-TEV-hsNUP98$_{FG\ 1\text{-}505,\ \Delta GLEBS}^{A221C}$	Fig.1-3, Extended data Fig.3, 8-10
pQE-14His-TEV-hsNUP98$_{FG\ 1\text{-}505,\ \Delta GLEBS}^{A221C\text{-}S244C}$	Fig. 3, Extended data Fig.8-10
pQE-14His-TEV-hsNUP98$_{FG\ 1\text{-}505,\ \Delta GLEBS}^{A221C\text{-}S312C}$	Fig. 3, Extended data Fig.8-10
pQE-14His-TEV-hsNUP98$_{FG\ 1\text{-}505,\ \Delta GLEBS}^{A221C\text{-}S362C}$	Fig. 3, Extended data Fig.8-10
pQE-14His-TEV-hsNUP98$_{FG\ 1\text{-}505,\ \Delta GLEBS}^{A221C\text{-}A426C}$	Fig. 3, Extended data Fig.8-10
pQE-14His-TEV-hsNUP98$_{FG\ 1\text{-}505,\ \Delta GLEBS}^{A221C\text{-}A458C}$	Fig. 3, Extended data Fig.8-10
pQE-14His-TEV-hsNUP98$_{FG\ 1\text{-}505,\ \Delta GLEBS}^{A221C\text{-}A482C}$	Fig. 3, Extended data Fig.8-10
pQE-14His-TEV-hsNUP98$_{FG\ 1\text{-}505}$	Extended data Fig.10
pQE-14His-TEV-hsNUP98$_{FG\ 1\text{-}505}^{A221C\text{-}S244C}$	Extended data Fig.10
pQE-14His-TEV-hsNUP98$_{FG\ 1\text{-}505}^{A221C\text{-}S312C}$	Extended data Fig.10

pQE-14His-TEV-hsNUP98$_{FG\ 1\text{-}505}^{A221C\text{-}S362C}$	Extended data Fig.10
pQE-14His-TEV-hsNUP98$_{FG\ 1\text{-}505}^{A221C\text{-}A458C}$	Extended data Fig.10
pQE-14His-TEV-hsNUP98$_{FG\ 1\text{-}505}^{A221C\text{-}A482C}$	Extended data Fig.10

Supplementary Table 2. List of the FG-NUPs and their grafting sites in NPC model I. Dynamic and frozen regions have type numbers >1 and =1, respectively. The equation of motion of the frozen regions is not integrated during the simulation while the equation of motion of the dynamic regions is integrated. In order to distinguish between different FG-NUPs, different types are assigned, although all interaction parameters are identical for all FG-NUPs. The 3D coordinates of all NUPs with their "mol" and "type" numbers are provided in a supplementary data file (NPC_Model_I_homopolymer.lammpstrj), where "mol" numbers distinguish individual chains and "type" numbers identify the dynamic chains of distinct sequence type (e.g., NUP54, NUP58, …). Dynamic and frozen regions in the model have type numbers >1 and =1, respectively. Frozen regions effectively have infinite mass, i.e., they interact with all dynamic regions but they do not move. The "grafting residue" is the residue anchoring the chain to the NPC scaffold and is thus part of the frozen region.

Name of NUP	Number of copies	Beginning/ending residues	Type number of dynamic region in coordinate file	Type number of frozen region in coordinate file	Grafting residues of dynamic region(s)	Frozen residue number range
NUP54	32	2-493	2	1	PRO111	111-493
NUP58	32	2-599	3	1	ASN246, LEU418	246-418
NUP62	40	2-502	4	1	ALA331	331-502
NUP98	48	2-615	6	1	LYS595	595-615
NUP214	8	700-2090	5	1	SER974	700-974
NUP358	40	4-3224	9	1	SER757	1-757
Scaffold NUPs	-	-	-	1	-	all

*Not included in model I are NUP153 and Pom121.

Supplementary Table 3. List of the FG-NUPs and their grafting sites in NPC model II. Dynamic and frozen regions have type numbers >1 and =1, respectively. The equation of motion of the frozen regions is not integrated during the simulation while the equation of motion of the dynamic regions is integrated. In order to distinguish between different FG-NUPs, different types are assigned, although all interaction parameters are identical for all FG-NUPs. The coordinates of all NUPs with "mol" and "type" numbers are provided in a supplementary data file (NPC_Model_II_homopolymer.lammpstrj), where "mol" numbers distinguish individual chains and "type" numbers identify the dynamic chains of distinct sequence type (e.g., NUP54, NUP58, …). Dynamic and frozen regions in the model have type numbers >1 and =1, respectively. Frozen regions effectively have infinite mass, i.e., they interact with all dynamic regions but they do not move. The "grafting residue" is the residue anchoring the chain to the NPC scaffold and is thus part of the frozen region.

Name of NUP	Number of copies	Beginning/ending residues	Type number of dynamic region in coordinate file	Type number of frozen region in coordinate file	Grafting residues of dynamic region(s)	Frozen residue number range
NUP54	32	2-493	2	1	PRO111	111-493
NUP58	32	2-599	3	1	ASN246, LEU418	246-418
NUP62	48	2-502	4	1	ALA331	331-502
NUP98	48	2-615	6	1	LYS595	595-615
POM121	16	2-1249	8	1	GLU72	2-72
NUP153	16	272-1475	7	1	ALA272	272
NUP214	8	700-2090	5	1	SER974	700-974
NUP214	8	954-2090	5	1	ARG954	954
NUP358	40	4-3224	9	1	SER757	1-757
Scaffold NUPs	-	-	-	1	-	all

Supplementary Table 4. List of parameters used in NPC model I with explicit solvent. The cross-interaction parameters between frozen beads i.e., scaffold residues and membrane beads, are not given.

Type of particle	Scaffold residue ($j \in p_{sc}$)	FG residue ($j \in p_{FG}$)	Membrane ($j \in m$)	Solvent $j \in s$
Scaffold residue ($i \in p_{sc}$)	-	$\sigma_{ij} = \sigma$, $r_c = 2\sigma$, $\tilde{\epsilon}_{ij} = 0.1$	-	$\sigma_{ij} = 0.783\ \sigma$, $r_c = 0.8788\sigma$, $\tilde{\epsilon}_{ij} = 0.1$
FG residue ($i \in p_{FG}$)	Symmetric	$\sigma_{ij} = \sigma$, $r_c = 2\sigma$, $\tilde{\epsilon}_{ij} = \tilde{\epsilon}$	$\sigma_{ij} = 1.78\sigma$, $r_c = 1.99798\sigma$, $\tilde{\epsilon}_{ij} = 0.1$	$\sigma_{ij} = 1.78\sigma$, $r_c = 1.99798\sigma$, $\tilde{\epsilon}_{ij} = 0.1$
Membrane ($i \in m$)	-	Symmetric	-	$\sigma_{ij} = 1.78\sigma$, $r_c = 1.99798\sigma$, $\tilde{\epsilon}_{ij} = 0.1$
Solvent ($i \in s$)	Symmetric	Symmetric	Symmetric	$\sigma_{ij} = 0.783\ \sigma$, $r_c = 0.8788\sigma$, $\tilde{\epsilon}_{ij} = 0.1$

Supplementary references

86. Lakowicz, J. R., Gryczyński, I., Wiczk, W., Kuśba, J. & Johnson, M. L. Correction for incomplete labeling in the measurement of distance distributions by frequency-domain fluorometry. *Anal. Biochem.* **195**, 243–254 (1991).
87. Flory, P. J. Thermodynamics of high polymer solutions. *The Journal of chemical physics* **10**, 51–61 (1942).
88. Huggins, M. L. Solutions of long chain compounds. *The Journal of chemical physics* **9**, 440 (1941).
89. de Gennes, P.-G. *Scaling concepts in polymer physics*. (Cornell university press, 1979).
90. Weeks, J. D., Chandler, D. & Andersen, H. C. Role of repulsive forces in determining the equilibrium structure of simple liquids. *J. Chem. Phys.* **54**, 5237–5247 (1971).
91. Boyd, K. J. & May, E. R. BUMPy: A Model-Independent Tool for Constructing Lipid Bilayers of Varying Curvature and Composition. *J. Chem. Theory Comput.* **14**, 6642–6652 (2018).
92. Wassenaar, T. A., Ingólfsson, H. I., Böckmann, R. A., Tieleman, D. P. & Marrink, S. J. Computational Lipidomics with *insane* : A Versatile Tool for Generating Custom Membranes for Molecular Simulations. *J. Chem. Theory Comput.* **11**, 2144–2155 (2015).
93. Tran, H. T. & Pappu, R. V. Toward an accurate theoretical framework for describing ensembles for proteins under strongly denaturing conditions. *Biophys. J.* **91**, 1868–1886 (2006).
