# Supplementary material for: Visualizing the disordered nuclear transport machinery in situ
Source: Nature. 2023 Apr 26;617(7959):162–9. doi: 10.1038/s41586-023-05990-0 (PMC10156602; doi:10.1038/s41586-023-05990-0)
Supplement: Supplementary file 2 — Reporting Summary [file 41586_2023_5990_MOESM2_ESM.pdf]

nature portfolio

Corresponding author(s): Edward Lemke & Gerhard Hummer

Last updated by author(s): Feb 17, 2023

Reporting Summary

Nature Portfolio wishes to improve the reproducibility of the work that we publish. This form provides structure for consistency and transparency in reporting. For further information on Nature Portfolio policies, see our Editorial Policies and the Editorial Policy Checklist.

Statistics

For all statistical analyses, confirm that the following items are present in the figure legend, table legend, main text, or Methods section.

n/a	Confirmed	
☐	☒	The exact sample size (*n*) for each experimental group/condition, given as a discrete number and unit of measurement
☐	☒	A statement on whether measurements were taken from distinct samples or whether the same sample was measured repeatedly
☐	☒	The statistical test(s) used AND whether they are one- or two-sided<br>*Only common tests should be described solely by name; describe more complex techniques in the Methods section.*
☐	☒	A description of all covariates tested
☐	☒	A description of any assumptions or corrections, such as tests of normality and adjustment for multiple comparisons
☐	☒	A full description of the statistical parameters including central tendency (e.g. means) or other basic estimates (e.g. regression coefficient) AND variation (e.g. standard deviation) or associated estimates of uncertainty (e.g. confidence intervals)
☐	☒	For null hypothesis testing, the test statistic (e.g. *F*, *t*, *r*) with confidence intervals, effect sizes, degrees of freedom and *P* value noted<br>*Give P values as exact values whenever suitable.*
☒	☐	For Bayesian analysis, information on the choice of priors and Markov chain Monte Carlo settings
☒	☐	For hierarchical and complex designs, identification of the appropriate level for tests and full reporting of outcomes
☐	☒	Estimates of effect sizes (e.g. Cohen's *d*, Pearson's *r*), indicating how they were calculated

Our web collection on statistics for biologists contains articles on many of the points above.

Software and code

Policy information about availability of computer code

Data collection	Symphotime v2.6
Data analysis	Symphotime v2.6, PAM-PIE(https://gitlab.com/PAM-PIE/PAM), Matlab R2021a, LAMMPS(stable version 29Sep2021-Update3) software package, GROMACS(version 2020.6), VMD(version 1.9.3).

For manuscripts utilizing custom algorithms or software that are central to the research but not yet described in published literature, software must be made available to editors and reviewers. We strongly encourage code deposition in a community repository (e.g. GitHub). See the Nature Portfolio guidelines for submitting code & software for further information.

Data

Policy information about availability of data

All manuscripts must include a data availability statement. This statement should provide the following information, where applicable:

- Accession codes, unique identifiers, or web links for publicly available datasets
- A description of any restrictions on data availability
- For clinical datasets or third party data, please ensure that the statement adheres to our policy

The NPC scaffold used in the MD simulation is PDB ID: 7R5K. The source data for the main and extended data figures as well as coordinate for MD simulations are provided in supplementary information. Initial configurations and trajectories of the molecular dynamics simulations are available at https://doi.org/10.5281/zenodo.7648957 under CC-BY license. All other data are available in the main text or supplementary information.

Human research participants

Policy information about studies involving human research participants and Sex and Gender in Research.

Reporting on sex and gender	NA
Population characteristics	NA
Recruitment	NA
Ethics oversight	NA

Note that full information on the approval of the study protocol must also be provided in the manuscript.

Field-specific reporting

Please select the one below that is the best fit for your research. If you are not sure, read the appropriate sections before making your selection.

☒ Life sciences ☐ Behavioural & social sciences ☐ Ecological, evolutionary & environmental sciences

For a reference copy of the document with all sections, see nature.com/documents/nr-reporting-summary-flat.pdf

Life sciences study design

All studies must disclose on these points even when the disclosure is negative.

Sample size	The sample size (~100 cells per mutant) in FLIM-FRET measurements was chosen so that the total photon numbers are sufficient enough for a very robust fitting of the fluorescent lifetime decay of together 18 mutants.
Data exclusions	No data were excluded from the analyses.
Replication	The transport assay of labelled cells and reconstituted in vitro condensates was repeated 3 times independently with same conclusion. The acceptor photobleaching assay to check intermolecular and intramolecular FRET was repeated 5 times independently with same conclusion. All attempts at replication were successful.
Randomization	Cells were seeded, transfected and labelled in imaging dishes, and the red fluorescent channel was used to guide the eye to identify cells with a fluorescently labelled nuclear rim. After image acquisition, a threshold was applied (as explained in Methods) to exclude cells with too high expression levels from further analysis. No further randomization was applied, all measured cells were analysed.
Blinding	The investigators were blinded to group allocation during data collection and analysis.

Reporting for specific materials, systems and methods

We require information from authors about some types of materials, experimental systems and methods used in many studies. Here, indicate whether each material, system or method listed is relevant to your study. If you are not sure if a list item applies to your research, read the appropriate section before selecting a response.

Materials & experimental systems

n/a	Involved in the study	
☐	☒	Antibodies
☐	☒	Eukaryotic cell lines
☒	☐	Palaeontology and archaeology
☒	☐	Animals and other organisms
☒	☐	Clinical data
☒	☐	Dual use research of concern

Methods

n/a	Involved in the study	
☒	☐	ChIP-seq
☒	☐	Flow cytometry
☒	☐	MRI-based neuroimaging

Antibodies

Antibodies used	Anti-KPNB1 antibody (Abcam, ab2811) and anti-mouse-Alexa Fluor 488 secondary antibody (ThermoFisher, A-11001)
Validation	Antibodies were validated by the manufactures (for Anti-KPNB1, see https://www.abcam.com/kpnb1-antibody-3e9-ab2811.pdf; for

Validation	anti-mouse-AF488, see https://www.thermofisher.com/antibody/product/Goat-anti-Mouse-IgG-H-L-Cross-Adsorbed-Secondary-Antibody-Polyclonal/A-11001).

Eukaryotic cell lines

Policy information about cell lines and Sex and Gender in Research

Cell line source(s)	COS-7 (Sigma 87021302)
Authentication	Authenticated by the manufacturer. Validation by morphology.
Mycoplasma contamination	All cell lines were regularly tested for mycoplasma contamination, with negative results.
Commonly misidentified lines (See ICLAC register)	No misindentified lines were used.
